# Predictors of cognitive resilience in the old‐old: An observational study using real‐world electronic health record data

**DOI:** 10.1002/alz.71443

**Published:** 2026-05-01

**Authors:** Konstantina Skolariki, Paul B. Rosenberg, Esther S. Oh, Jeannie Leoutsakos, Constantine G. Lyketsos, Roy Adams

**Affiliations:** ^1^ Department of Psychiatry and Behavioral Sciences Johns Hopkins University School of Medicine Baltimore Maryland USA; ^2^ Richman Family Precision Medicine Center of Excellence in Alzheimer's Disease Johns Hopkins University Baltimore Maryland USA; ^3^ Division of Geriatric Medicine and Gerontology Department of Medicine Johns Hopkins University School of Medicine Baltimore Maryland USA; ^4^ Department of Computer Science Johns Hopkins University Baltimore Maryland USA

**Keywords:** aging, cognitive resilience, EHR data, old‐old, predictors

## Abstract

**INTRODUCTION:**

Understanding factors associated with cognitive resilience is critical for developing interventions to preserve cognitive health in the old‐old. Our aim is identifying clinical predictors of cognitive resilience in a large real‐world cohort of adults aged 75+.

**METHODS:**

Longitudinal analysis of electronic health records from Johns Hopkins primary care clinics between January 1, 2014, and August 31, 2025. A minimum of two follow‐up visits at least a year apart were required. To estimate the associations between predictors and cognitive resilience, we employed a Cox model with cognitive disorder diagnosis as the outcome.

**RESULTS:**

Of 43,178 patients, 6563 developed cognitive disorder during follow‐up. Psychiatric conditions were strongly associated with decreased resilience. Antihypertensive use was associated with increased resilience. Black race and Medicaid enrollment were associated with decreased resilience.

**DISCUSSION:**

Most adults reaching 90 remained cognitively healthy. Resilience reflected physiological reserve and care engagement, while vascular, psychiatric, and social factors reduced resilience.

## Highlights

- Electronic health record (EHR) data from 43,178 patients used to assess 81 predictors and their association with cognitive resilience in the old‐old.
- Psychiatric conditions, including schizophrenia, bipolar disorder, and depression, were strongly associated with decreased cognitive resilience.
- Antihypertensive medication use was associated with increased resilience.
- Psychotropic medication use was associated with decreased resilience.
- Black race and Medicaid enrollment were associated with decreased resilience.

## 1 BACKGROUND

As the global population ages at an unprecedented rate, with the “old‐old” (adults aged 75 and older) representing the fastest‐growing demographic worldwide,^1^ the identification of individuals who successfully age without developing cognitive disorders has become a critical area of research. By 2050, the number of those aged 75+ is projected to exceed 426 million globally, creating an urgent imperative to understand mechanisms underlying cognitive preservation. Cognitive resilience—the remarkable capacity to maintain cognitive function despite the multifaceted biological, neurological, and physiological challenges associated with advanced age—remains one of the most poorly understood yet consequential phenomena in gerontological research, particularly in the understudied old‐old. This study aims to examine the clinical and biological factors that contribute to healthy cognitive aging within this age group, utilizing a large, real‐world dataset derived from the primary care electronic health records (EHRs) of Johns Hopkins Community Physicians. By studying a cohort of individuals who reached age 75 without dementia or mild cognitive impairment (MCI), we seek to identify key predictors of cognitive resilience, including demographic variables, chronic comorbidities, laboratory results, vital signs, and medication history. This prospective cohort study integrates longitudinal data to examine the relationships between these factors and the likelihood of remaining cognitively healthy in advanced age. The findings will provide valuable insights into the clinical phenotypes associated with cognitive resilience, potentially informing early interventions aimed at preserving cognitive function in the oldest population.

## 2 METHODS

### 2.1 Data source and population

To examine factors associated with cognitive resilience in the old old we included EHRs from all patients who had at least one cognitive disorder free clinical visit at or after age 75 or older in the Johns Hopkins primary care clinics between January 1, 2014, and August 31, 2025. Time zero was defined as the first visit after age 75 in patients with no prior diagnosis of cognitive disorder. We included data only from patients who had a minimum of two visits at least one year apart after time zero. A minimum of two visits were required to establish a follow‐up interval over which cognitive disorder onset could be assessed. Potential predictors of resilience included demographics (age, gender, race, Medicaid enrollment, and hospital visits), psychiatric and nonpsychiatric chronic conditions reported in the Chronic Conditions Data Warehouse (see Table S1), laboratory measurements (see Table S2), vital signs (blood pressure [BP] measurements and body mass index [BMI]), and medications (BP medications, cholesterol medications, psychotropics, anticoagulants). Laboratory measurements, medication orders, and vital signs recorded from time zero to before any cognitive disorder diagnosis were included as continuous variables. Participants with no follow‐up visit after time zero were excluded (Figure S1). Patients with a cognitive disorder diagnosis prior to time zero visit were also excluded. This study was approved by a Johns Hopkins Institutional Review Board (IRB00228485 and IRB00269466). A waiver of consent was granted as the data were collected during routine care, without any modifications to standard care. Appropriate safeguards were in place to minimize the risk of privacy breaches.

### 2.2 Outcome

#### 2.2.1 Outcome classification

The primary outcome was time to incident cognitive disorder after age 75 (i.e. dementia or MCI, collectively referred to as a cognitive disorder). Diagnoses were identified based on the presence of one or more encounter diagnoses or hospital billing International Classification of Diseasess 10^th^ Revision (ICD‐10) codes, as detailed in Table S1. The timing of the outcome was defined as the date of first cognitive disorders diagnosis after time zero in the patient's EHR. ICD‐10 codes used to identify dementia included F01.XX, F02.XX, F03.XX, G30.XX, G31.0X, G31.1, and G31.83 while code G31.84 was used to identify MCI. Dementia subtypes (e.g. Alzheimer's) were not differentiated in this analysis. Only diagnoses of a cognitive disorder made after time zero were captured and included in the analysis. Participants were classified as cognitively resilient if they had at least one clinical visit at or after age 90 without a cognitive disorder diagnosis.

### 2.3 Features

#### 2.3.1 Demographics

Age, gender, and race were incorporated as covariates in our analysis. Age is a key risk factor for cognitive disorders,^2^ and sex differences have been observed in the incidence of cognitive disorders.^3^, ^4^ Race was included due to established racial disparities in the prevalence of cognitive disorders.^5^ Self‐reported race was categorized as American Indian, Asian, Black, Pacific Islander, White, unknown, or “other” when no further details were provided, while Hispanic versus non‐Hispanic ethnicity was determined from clinical intake forms. Medicaid enrollment was included as a proxy to socioeconomic status. Number of hospital visits was also included as a feature. Education information was excluded due to incomplete data in the cohort. Zip code was not available in a nine‐digit zip code and was likewise not included.^6^

#### 2.3.2 Vitals, lab results and medication orders

Results of laboratory studies (see Table S2), and vital signs (BP measurements and BMI), recorded from time zero and before any cognitive disorder diagnosis were included. Median values were used for longitudinal laboratory and vital sign measurements. Medication orders (BP medications, cholesterol medications, psychotropics, anticoagulants) (Table S3) recorded anytime were included and categorized as binary (no medications, and on medications).

#### 2.3.3 Comorbidities

We included multiple comorbidities as identified in the Chronic Conditions Warehouse by ICD‐10 diagnosis codes^7^ (Table S1). Such diagnoses documented during any clinical visit prior to a cognitive disorder diagnosis, or turning 90 without a cognitive disorder diagnosis, were included in the data analysis. Chronic conditions were treated as binary covariates.

### 2.4 Statistical analysis

#### 2.4.1 Primary analysis

To estimate the associations between baseline predictors and cognitive resilience, we employed a Cox model with cognitive disorder diagnosis as the outcome event. Time‐to‐event was calculated from the date of first visit at age 75 without cognitive disorder to either: (1) cognitive disorder diagnosis, (2) death, or (3) censoring. To facilitate interpretation of factors associated with cognitive resilience (successfully reaching age 90 without cognitive disorder), we inverted the hazard ratios (HRs) into resilience ratios (RRs). Under this adjustment, RRs greater than 1.0 indicate increased likelihood of cognitive resilience, while values less than 1.0 indicate decreased likelihood of resilience. To account for the informative nature of missing laboratory measurements, we implemented a missing indicator approach. For each laboratory variable with missing values, we created a binary indicator variable prior to imputation. The actual laboratory values were then imputed using median values to maintain model stability. As this analysis is observational in nature, the associations identified should not be interpreted as causal relationships but rather as hypothesis generating associations between predictors and the outcome. To account for multiple testing across the predictors, we applied Bonferroni correction. *p*‐values were calculated using a two‐tailed Wald test with significance set at *p* < 0.05. All analyses were conducted in Python using the lifelines package, version 0.30.0.

**RESEARCH IN CONTEXT**

1. **Systematic review**: We analyzed electronic health records from a large primary care cohort of 43,178 patients aged 75 and older, complemented by a comprehensive review of the existing literature on predictors of cognitive resilience in the old‐old.
2. **Interpretation**: The findings contribute to our understanding of factors associated with cognitive resilience (surviving to age 90 without a cognitive disorder diagnosis). Psychiatric conditions, including schizophrenia, bipolar disorder, and depression, were the strongest predictors of decreased resilience. Antihypertensive medication use was the only medication class associated with increased cognitive resilience.
3. **Future directions**: Future research should focus on determining whether interventions targeting psychiatric conditions and blood pressure control can improve cognitive outcomes in older adults. Randomized trials and causal inference methods are needed to distinguish direct medication effects from confounding by indication.

#### 2.4.2 Resilience curves

For this study, cognitive resilience is defined as survival to age 90 without a diagnosis of a cognitive disorder. Resilience curves were generated using Kaplan–Meier estimators and were stratified by gender (female, male, overall) as well as by race (Black, non‐Black, overall), with death treated as censoring. These curves show the likelihood of surviving to 90 without having a cognitive disorder diagnosis (cognitive resilience). Log‐rank tests were used to assess statistical differences between groups.

#### 2.4.3 Medication analysis

To examine the effect of medications on cognitive resilience, we categorized medication orders relative to age 75 (time zero) into two groups: (1) no medication (reference), and (2) on medication(s). Four medication classes were examined: antihypertensives, cholesterol agents, psychotropics, and anticoagulants. We fit a Cox model and RR computed as the inverse of the hazard ratio (RR = 1/HR). Values greater than 1 indicate higher likelihood of maintaining cognitive resilience, while values less than 1 indicate lower likelihood. These associations should be interpreted as observational findings: medication initiation may also reflect underlying disease burden rather than a causal effect on cognitive decline.

#### 2.4.4 Sensitivity and secondary and analyses

We repeated the primary analysis using Fine–Gray subdistribution hazard models to account for death as a competing risk (Figure S2). An additional sensitivity analysis was performed, restricting our cohort into a subsample that only included individuals living in Baltimore city to account for individuals closer to the healthcare system. In secondary analyses, we examined univariate associations between individual predictors and cognitive resilience using Cox models. Furthermore, in an additional secondary analysis we stratified participants based on hypertension diagnosis (hypertension diagnosis v. no hypertension diagnosis), BP medication duration (on BP meds < 5 years, vs. on BP meds ≥ 5 years, vs. no BP meds), and median systolic BP (SBP) categories (< 120 mmHg, vs. 120–139 mmHg, vs. ≥140 mmHg).^8^ Lastly, we stratified participants based on psychiatric diagnosis and psychotropic medications (no psychiatric diagnosis, vs. no psychiatric and on psychotropic meds, vs. psychiatric diagnosis and no psychotropic meds, vs. psychiatric diagnosis and psychotropic meds).

## 3 RESULTS

### 3.1 Sample characteristics

From an initial sample of 168,490 patients, a total of 43,178 patients aged 75 years met our inclusion criteria, of whom 6489 later developed a cognitive disorder (Figure S1). Sample characteristics are in Table 1. 6386 patients survived to age 90, 68% survived to age 90 without a cognitive disorder diagnosis. The median age at time zero was 75.1 (interquartile range [IQR] 75.0–76.0), 25,116 patients were women (58.2%), and 12,254 (28.4%) were non‐white. The median (IQR) number of healthcare visits was 96 (31–204). Significant differences, among cognitively resilient patients and patients with a cognitive disorder diagnosis, regarding psychiatric and nonpsychiatric comorbidities. More cognitively resilient patients had been diagnosed with nonpsychiatric conditions (i.e. hypertension: 79.4% vs. 28.6%, Atrial fibrillation 18.5% vs. 8.0%).

**TABLE 1:** Patients’ characteristics.

Characteristic	Overall	Censored	Surviving to 90 w/o cognitive disorders	Surviving to 90 w cognitive disorders	*p*‐value
*N*, (%)	43,178	36,792	4341	2045	–
Age, y, median (IQR)	75.1 (75.0–76.0)	75.1 (75.0–75.5)	78.0 (75.3–83.7)	78.2 (75.4–84.1)	< 0.001
Female, *N* (%)	25,116 (58.2)	21,039 (57.2)	2689 (62.0)	1388 (67.9)	< 0.001
Race, *N* (%)					
White	30,924 (71.6)	26,210 (71.2)	3269 (75.3)	1445 (70.7)	< 0.001
Black	7508 (17.4)	6449 (17.5)	646 (14.9)	413 (20.2)	
Asian	995 (2.3)	815 (2.2)	121 (2.8)	59 (2.9)	
Other	2018 (4.7)	1795 (4.9)	147 (3.4)	76 (3.7)	
Unknown	1733 (4.0)	1523 (4.1)	158 (3.6)	52 (2.5)	
Hispanic ethnicity, *N* (%)	1019 (2.4)	876 (2.4)	85 (2.0)	58 (2.8)	0.08
Comorbidities, *N*, (%)					
Hyperlipidemia	31,002 (71.8)	29,526 (80.3)	910 (21.0)	566 (27.7)	< 0.001
Hypertension	28,363 (65.7)	27,252 (74.1)	768 (17.7)	343 (16.8)	< 0.001
Diabetes	11,975 (27.7)	11,424 (31.05)	358 (8.3)	193 (9.4)	< 0.001
Ischemic heart disease	11,856 (27.5)	11,012 (29.9)	515 (11.9)	329 (16.1)	< 0.001
Chronic kidney disease	10,669 (24.7)	9865 (26.8)	479 (11.0)	325 (15.9)	< 0.001
Atrial fibrillation	7321 (17.0)	6732 (18.3)	396 (9.1)	193 (9.4)	< 0.001
Stroke/TIA	6208 (14.4)	5533 (15.0)	346 (8.0)	329 (16.1)	< 0.001
HF/non‐IHD	6451 (14.9)	5867 (16.0)	378 (8.7)	206 (10.1)	< 0.001
Anxiety	12,732 (29.5)	11,329 (30,8)	775 (17.9)	628 (30.7)	< 0.001
Depression	11,390 (26.4)	10,520 (28.6)	478 (11.0)	392 (19.2)	< 0.001
Bipolar disorder	1364 (3.2)	1250 (3.4)	44 (1.0)	70 (3.4)	< 0.001
Alcohol use disorder	1350	1337	11	2	0.26
ADHD	533	529	2	2	0.75
Schizophrenia	638 (1.5)	524 (1.4)	31 (0.7)	83 (4.1)	< 0.001
Cognitive disorders	6489 (15.0)	4444 (12.1)	0	2045 (100)	
Follow‐up, y, median (IQR)	5.3 (2.3–9.6)	4.4 (1.9–7.8)	15.3 (10.5–17.5)	15.3 (11.1–17.9)	< 0.001
Mortality, *N*, (%)	10,301 (23.9)	6998 (19.0)	2063 (47.5)	1240 (60.6)	< 0.001
Hospital visits/year, median^*^ (IQR)	38.9 (19.8–86.7)	40.8 (20.2–92.3)	19.6 (9.8–33.6)	16.6 (9.9–28.4)	< 0.001

Abbreviations: ADHD, attention deficit hyperactivity disorder; HF, heart failure; IQR, interquartile range; N, numbers; non‐IHD, non‐ischemic heart disease; TIA, transient ischemic attack; y, years.

^*^Median number of visits per patient.

In our analysis of 81 predictors (medications, laboratory measures, vitals, diagnosis, hospital visits count, and demographics), we identified 24 factors that were statistically significantly associated with either increased or decreased likelihood of cognitive resilience. Fine – Gray analyses did not significantly alter these estimates (Figure S2). The following effect estimates remained similar in direction in the univariate analysis (Table S4).

### 3.2 Psychiatric conditions were linked with decreased cognitive resilience

**FIGURE 1:**
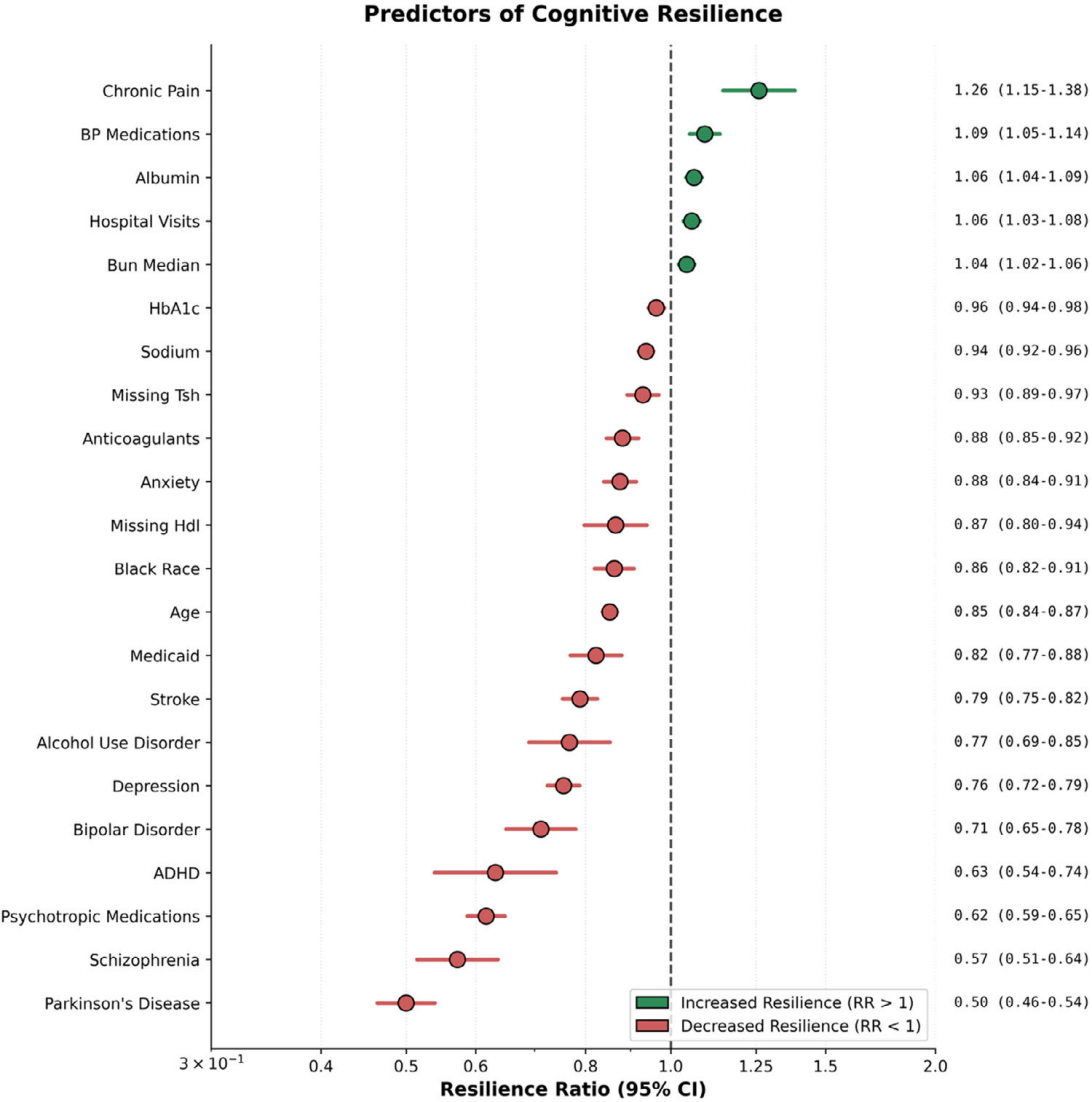
All statistically significant predictors from multivariate analysis (*p* < 0.001).

The Cox model showed several psychiatric comorbidities (Figure 1) to be strongly associated with lower likelihood of cognitive resilience. These conditions include bipolar disorder (RR, 0.72; 95% confidence interval [CI], 0.66–0.79; *p* < 0.001), attention deficit hyperactivity disorder (ADHD; RR, 0.64; 95% CI, 0.55–0.76; *p* < 0.001), depression (RR, 0.74; 95% CI, 0.71–0.78; *p* < 0.001), anxiety (RR, 0.87; 95% CI, 0.83–0.90; *p* < 0.001), alcohol use disorder (AUD; RR, 0.77; 95% CI, 0.70–0.86; *p* < 0.001), and schizophrenia (RR, 0.56; 95% CI, 0.50–0.62; *p* < 0.001).

### 3.3 Covariates associated with increased cognitive resilience

Having orders for BP medications was associated with increased resilience (RR, 1.09; 95% CI 1.05–1.14; *p* < 0.001). Chronic pain (RR, 1.26; 95% CI, 1.15–1.38; *p* < 0.001) and number of hospital visits (RR, 1.06; 95% CI, 1.03–1.08; *p* < 0.001) were also associated with increased resilience. Furthermore, higher blood albumin (RR, 1.06; 95% CI, 1.04–1.09; *p* < 0.001) and higher blood urea nitrogen (BUN) (RR, 1.04; 95% CI, 1.02–1.06; *p* < 0.001) were also associated with increased resilience.

### 3.4 Covariates associated with decreased cognitive resilience

Stroke was also associated with decreased resilience (RR, 0.79; 95% CI, 0.75–0.82, *p* < 0.001). Black race (RR, 0.86; 95% CI, 0.82–0.91; *p* < 0.001), higher HBA1C (RR, 0.96; 95% CI, 0.94–0.98; *p* < 0.001), and higher sodium (RR, 0.94; 95% CI, 0.92–0.96; *p* < 0.001) were associated with decreased cognitive resilience. Furthermore, being prescribed anticoagulants (RR, 0.88; 95% CI, 0.85–0.92, *p* < 0.001), and enrollment in Medicaid (RR, 0.82; 95% CI, 0.77–0.88; *p* < 0.001) were also associated with decreased resilience. A diagnosis or Parkinson's showed the strongest association with decreased resilience (RR, 0.50; 95% CI, 0.46–0.54; *p* < 0.001).

### 3.5 Cognitive resilience curves

**FIGURE 2:**
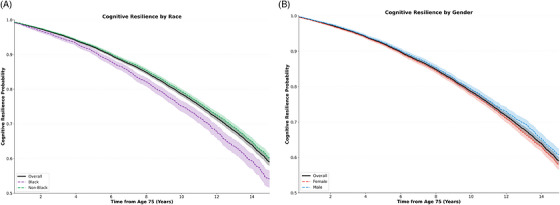
Cognitive resilience curves. Cognitive resilience curves (surviving to 90 with no cognitive disorders diagnosis) were stratified by gender (female v. male, and overall) as well as by race (Black, v. non‐Black, and overall). Shaded areas indicate 95% CIs. CI, confidence interval.

Cognitive resilience curves stratified by race (Black v. non–Black, and overall), and gender (female vs. male, and overall) are in Figure 2. Compared to Black individuals, non‐Black individuals had higher resilience (*p* < 0.001). Men had slightly higher cognitive resilience than women (*p* < 0.05).

### 3.6 Association between medication use and cognitive resilience

**FIGURE 3:**
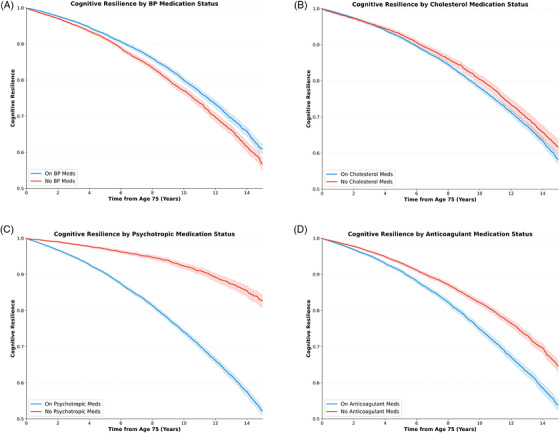
Cognitive resilience curves (surviving to 90 with no cognitive disorders diagnosis) stratified by medication status (on medication vs not on medication) for BP medication (A), cholesterol medication (B), psychotropic medication (C), and anticoagulants (D). Shaded areas indicate 95% CIs. BP, blood pressure; CI, confidence interval.

**FIGURE 4:**
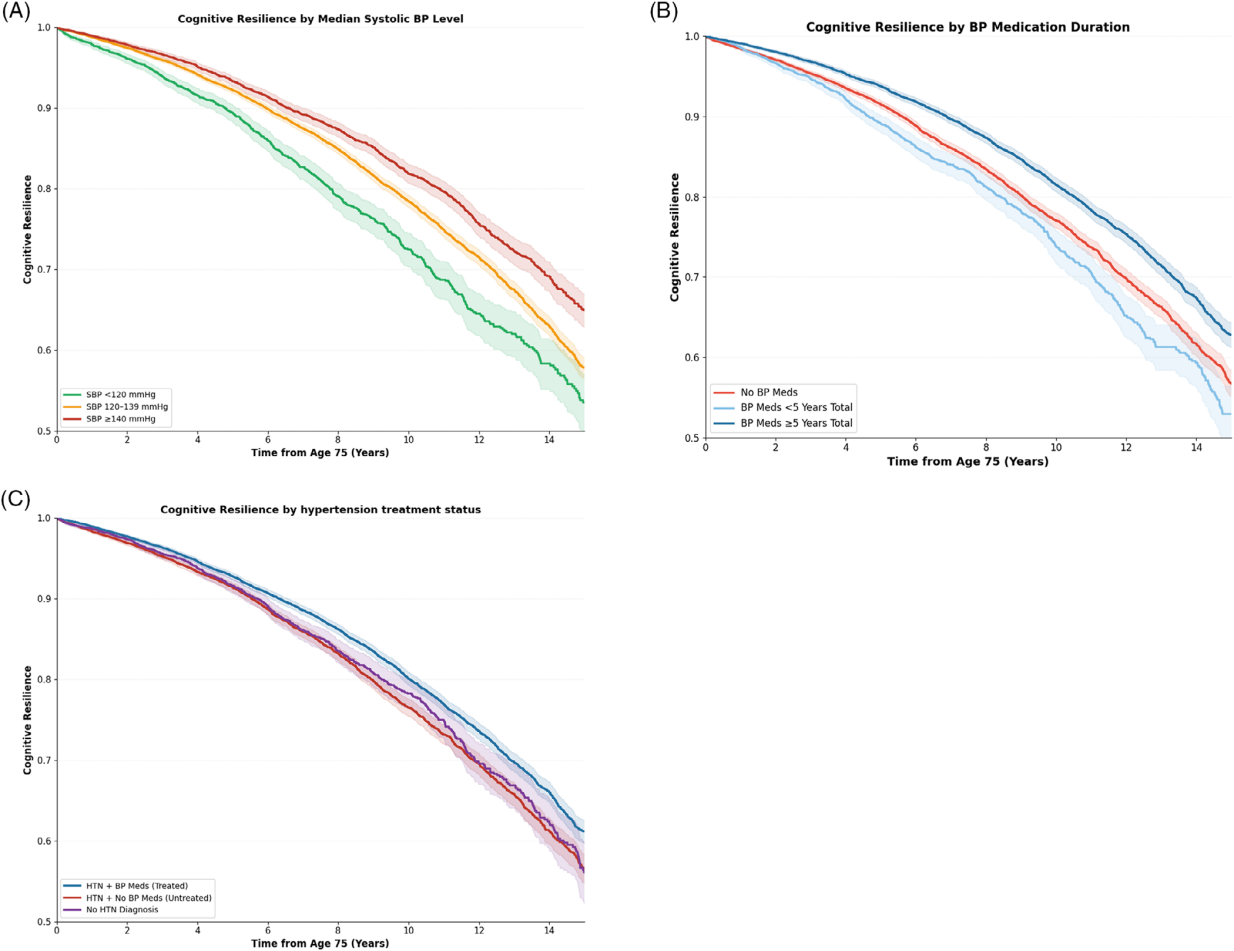
Cognitive resilience curves (surviving to 90 with no cognitive disorders diagnosis) stratified by median SBP levels (< 120 mmHg, 120–139 mmHg, and ≥ 140 mmHg) (A), BP medication duration (< 5 years, and ≥5 years) (B), and HTN treatment status (C). Shaded areas indicate 95% CIs. BP, blood pressure; CI, confidence interval; HRN, hypertension.

The association between medication use and cognitive resilience was examined for four medication classes (BP medication, cholesterol medication, psychotropic medication, and anticoagulants) (Figure 3). BP medication orders was the only medication class associated with increased cognitive resilience. Patients prescribed antihypertensive medications were significantly more likely to remain cognitively intact compared to non‐users (RR, 1.17; 95% CI: 1.11–1.23; *p* < 0.001). The association remained significant after multivariate adjustment (RR, 1.09, 95% CI, 1.05–1.14; *p* < 0.001). The stratified analysis showed that SBP ≥140 mmHg (RR, 1.21; 95% CI, 1.15–1.27; *p* < 0.001) (Figure 4A), being on BP meds for 5 or more years (RR, 1.14; 95% CI, 1.10–1.19; *p* < 0.001) (Figure 4B), and treated hypertension (RR, 1.07; 95% CI, 1.03–1.12; *p* = 0.001) were associated with increased resilience (Figure 4C).

**FIGURE 5:**
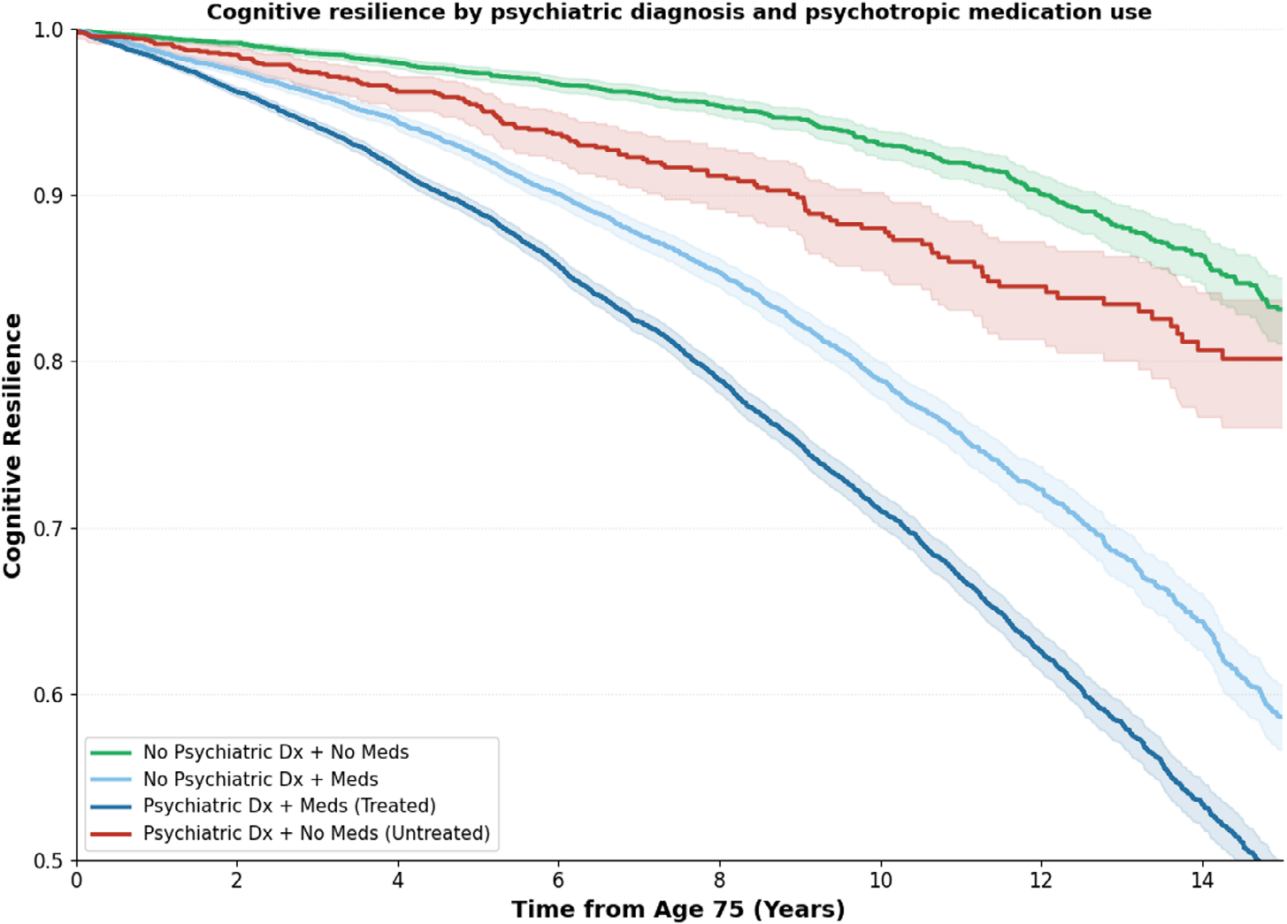
Cognitive resilience curves (surviving to 90 with no cognitive disorders diagnosis) stratified by psychiatric diagnosis and psychotropic medications. Shaded areas indicate 95% CIs. CI, confidence interval.

In contrast, psychotropic medication use was strongly associated with decreased cognitive resilience (RR, 0.29; 95% CI, 0.26–0.31; *p* < 0.001). Anticoagulant use (RR, 0.71; 95% CI, 0.67–0.74; *p* < 0.001) and cholesterol medication use (RR, 0.90; 95% CI, 0.85–0.96; *p* < 0.001) were also associated with decreased resilience. After multivariate adjustment, the associations for psychotropic medications (RR, 0.62; 95% CI, 0.59–0.66; *p* < 0.001), anticoagulants (RR, 0.88; 95% CI, 0.85–0.92; *p* < 0.001), and cholesterol medications (RR, 0.82; 95% CI, 0.78–0.87; *p* < 0.05) remained significant. The stratified analysis showed that individuals with no recorded psychiatric diagnosis but with psychotropic medication use (RR, 0.82; 95% CI, 0.78–0.86; *p* < 0.001) and individuals with both a psychiatric diagnosis and psychotropic medication use (RR, 0.59; 95% CI, 0.57–0.62; *p* < 0.001) showed decreased resilience (Figure 5).

### 3.7 Sensitivity analysis

Our sensitivity analysis into the subsample living in Baltimore city (*n* = 3161) showed that the majority of the features followed the same direction as seen in Figure 1 (Figure S3). BP medications (RR, 1.20; 95% CI, 1.03–1.40; *p* = 0.02), and albumin (RR, 1.08; 95% CI, 1.01–1.16; *p* = 0.03), were significantly associated with increased resilience. Parkinson's disease (RR, 0.56; 95% CI, 0.43–0.74; *p* < 0.001), schizophrenia (RR, 0.63; 95% CI, 0.46–0.86; *p* < 0.001), AUD (RR, 0.64; 95% CI, 0.48–0.84; < 0.001), psychotropic medications (RR, 0.70; 95% CI, 0.58–0.83; *p* < 0.001), cholesterol medications (RR, 0.78; 95% CI, 0.65–0.92; *p* < 0.001), stroke (RR, 0.78; 95% CI, 0.67–0.91; *p* < 0.001), and Medicaid (RR, 0.78; 95% CI, 0.65–0.93; *p* < 0.001) were significantly associated with decreased resilience. Two features showed the opposite direction in the Baltimore city subsample compared to our primary analysis. These two features were missing high density lipoprotein (HDL) lab results (RR, 1.08; 95% CI, 0.72–1.64; *p* = 0.71) and Black race (RR, 1.03; 95% CI, 0.89–1.19; *p* = 0.70).

## 4 DISCUSSION

We examined the association of various comorbidities, medications, and demographics with cognitive resilience in a “real‐world” data cohort of persons surviving to 90 years or older. Most patients (68%) who reached age 90 proceeded to remain free of cognitive disorders. Several covariates were associated with increased resilience while others were associated with decreased resilience. Chronic pain was associated with increased cognitive resilience. However, its’ very high prevalence (95%) and higher healthcare utilization (median visits 233 vs. 52) suggest this reflects healthcare engagement rather than a biological effect. On the other hand, stroke was associated with lower resilience as expected.^9^, ^10^ This likely reflects both pathological effects and increased surveillance. Hospital visit count showed a complex relationship with cognitive resilience. In multivariate Cox analysis, more visits were associated with increased resilience. However, event rates indicated that patients in the highest visit quartile had higher cognitive disorder rates (20.0%) compared to those in the lowest quartile (12.5%). This may reflect reverse causation, confounding, time‐dependent confounding, or selection effects. Patients in the preclinical stages of cognitive disorders may have increase healthcare utilization due to emerging symptoms before a formal diagnosis is recorded, meaning high visit counts may partly reflect impending cognitive disorder rather than predicting resilience. In addition, patients with more frequent clinical utilization have greater opportunity for cognitive disorders to be detected and documented, potentially inflating cognitive disorder rates in high‐utilization groups independent of true disease burden. Lastly, patients who survive longer naturally accumulate more visits. Selection effects related to geographical barriers, or healthcare engagement may differentially influence visit rates in ways that confound the association with resilience. These considerations suggest that the association between visit count and cognitive resilience should not be interpreted as reflecting a protective biological effect of healthcare engagement. Additionally, visit frequency may serve as a partial proxy for diagnostic surveillance intensity, as patients with more frequent healthcare contact have greater opportunity for cognitive symptoms to be observed and formally recorded. Under this interpretation, lower visit frequency may itself contribute to underdiagnosis of cognitive disorder, meaning that patients with lower healthcare engagement face greater risk of being misclassified as cognitively resilient when they may in fact have undetected cognitive disorders. This supports our broader concern that resilience may reflect documented rather than true cognitive health, with differential healthcare engagement contributing to misclassification.

Antihypertensive medication use was the only medication class associated with increased cognitive resilience. To address potential confounding by indication, we conducted stratified analyses by hypertension diagnosis, BP medication duration, and SBP categories. Treated hypertension was associated with increased resilience suggesting that the observed benefit reflects treatment rather than simply underlying disease burden. Furthermore, longer antihypertensive exposure (≥ 5 years) was associated with greater resilience, suggesting a duration‐dependent association that strengthens the case for a treatment effect.^11^, ^12^ However, antihypertensive use may also act as a proxy for healthcare engagement and longitudinal follow‐up, which could influence both treatment exposure and outcome ascertainment. As such, residual confounding, including confounding by indication, cannot be fully excluded. The association between SBP ≥ 140 mmHg and increased resilience calls for cautious interpretation. Individuals with persistently elevated SBP who survive to very old age may represent a selected, healthier subset. Furthermore, elevated SBP in the very old may reflect better cardiovascular reserve rather than pathological hypertension. This is consistent with our previous work showing that elevated SBP in older adults does not necessarily mean increased cognitive disorder risk, potentially due to competing mortality and the complex, age‐dependent relationship between BP and cognitive outcomes.^8^ These findings align with prior evidence suggesting benefits of BP control for cognitive outcomes.^13^, ^14^, ^15^, ^16^, ^17^, ^18^, ^19^, ^20^ The optimal BP threshold remains under investigation, though some studies suggest that keeping SBP around 130 mmHg may be more advantageous for older adults.^19^

In contrast, psychotropic medications had a strong negative association with cognitive resilience. This finding likely reflects confounding by indication rather than direct medication harm. Psychotropic medications are prescribed for psychiatric conditions which are themselves established risk factors for cognitive decline.^20^, ^21^, ^22^, ^23^, ^24^ In our analyses each of these psychiatric conditions were independently associated with decreased resilience, with schizophrenia and Parkinson's disease showing the strongest associations. The persistence of decreased resilience among individuals with psychotropic medication use but no recorded psychiatric diagnosis should be further explored. This group likely reflects incomplete diagnostic capture in EHR data. These findings suggest psychotropic medication use captures psychiatric burden beyond recorded diagnoses. Individuals with psychiatric diagnoses but no medication use showed higher resilience. This likely reflects a selection effect, where this subgroup disproportionately represents psychiatric conditions that did not require active pharmacological treatment, rather than indicating any protective effect of untreated psychiatric illness.

Anticoagulant use was associated with decreased resilience, likely reflecting underlying conditions such as atrial fibrillation.^25^, ^26^, ^27^ Cholesterol medication use showed a modest negative association, likely reflecting underlying cardiovascular disease burden.^27^, ^28^, ^29^ For anticoagulants and cholesterol medications, the underlying conditions necessitating treatment (i.e. atrial fibrillation, cardiovascular diseases, etc.) may exert stronger influences on cognition than BP. In contrast, hypertension represents a modifiable risk factor where treatment may confer benefit independent of disease‐related effects.^30^, ^31^ This could explain why antihypertensive use was associated with increased resilience, since BP control may intervene earlier in the pathophysiological cascade, whereas anticoagulants and cholesterol medication are often initiated after cardiovascular disease has already been diagnosed, when the cognitive burden of underlying vascular pathology may be less susceptible to intervention. We cannot distinguish direct medication effects from the effects of underlying conditions in this observational analysis. Future studies could focus on estimating the true effects of the medications on cognitive outcomes.

Elevated BUN was associated with greater resilience, likely reflecting survivorship rather than a direct protective effect.^32^, ^33^, ^34^, ^35^ Higher sodium was associated with decreased cognitive resilience in our cohort. This aligns with prior findings linking high sodium to cognitive decline.^36^, ^37^, ^38^

Cognitive resilience curves stratified by sex showed males with slightly higher resilience than females. However, this association reversed after adjusting for psychiatric conditions, reflecting a substantially higher prevalence of depression (37.1% vs. 25.5%) and anxiety (36.6% vs. 22.0%) in females. Additionally, males had higher mortality (26.3% vs. 21.4%), suggesting competing mortality contributes to the apparent male advantage.

Non‐Black individuals showed significantly higher cognitive resilience compared to Black individuals, with the latter demonstrating both higher cognitive disorder rates (18.3% vs. 14.4%) and higher mortality (24.7% vs. 23.8%). Similarly, Medicaid enrollment, included as a proxy for socioeconomic status, was independently associated with decreased resilience. These disparities are consistent with known health inequities and reflect differences in healthcare access, socioeconomic factors, and cumulative health burden not fully captured by our covariates.^39^, ^40^, ^41^ However, key social determinants, including education and neighborhood‐level deprivation indices, were unavailable or limited in our dataset, constricting causal interpretation and generalizability.^42^, ^43^ Lifetime socioeconomic trajectory could not be captured from primary care EHR data alone. Medicaid enrollment, while informative as a proxy, may limit causal interpretation. This limits both the causal interpretation of the racial and socioeconomic associations observed and the generalizability of findings beyond this healthcare system. Future studies integrating EHR and social determinants data are needed to better characterize these disparities. Our sensitivity analysis restricting the cohort to Baltimore city residents (*N* = 3161) partially addresses the geographic access component of this concern, seeing as patients living closer to healthcare access may be less likely to have cognitive disorder go undetected due to infrequent care contact. By restricting to this subgroup, we reduce the risk that low engagement driven by geographic barriers leads to systematic misclassification of patients as resilient. Most primary associations were preserved, suggesting geographic access does not explain the majority of findings. However, the opposite direction of the Black race association in this subsample, from being associated with decreased resilience in the full cohort to being associated with increased resilience in Baltimore city residents is noteworthy. This suggests that a component of the observed racial disparity in resilience reflects differential ascertainment bias rather than a true difference in underlying cognitive health, and that the primary analysis likely underestimates cognitive disorder burden in Black patients by misclassifying some individuals as resilient due to missed or delayed diagnoses. Residual differential ascertainment driven by clinician behavior, implicit bias, and under‐recognition of cognitive disorders in Black patients cannot be addressed through cohort restriction alone and is considered a limitation of this current work. Baltimore city is a specific urban environment with its own socioeconomic and demographic characteristics that differ from the broader cohort.

Study strengths include a large “real‐world” EHR dataset including a very large cohort of patients 90 years and older. We included 43,178 patients aged 75+ in the analysis, substantially exceeding the sample sizes of other US studies of the old‐old, which typically include around 1,000 participants.^44^, ^45^, ^46^ Limitations include the fact that these analyses are observational in nature, thus findings should be interpreted as hypothesis‐generating rather than causal. Additionally, diagnoses were based on routine clinical care rather than standardized criteria. The presence of a diagnosis is expected to be relatively accurate when recorded. However, the absence of a diagnosis is less informative, as many conditions may go unrecorded in primary care settings.

## 5 CONCLUSION

This large, real‐world observational study of 43,178 older adults age 75+ identified several clinical predictors of cognitive resilience after age 90. Psychiatric conditions were the strongest predictors of decreased resilience. These associations remained after multivariate adjustment. Antihypertensive medication was the only medication class associated with increased cognitive resilience, consistent with evidence that support the role of BP control in cognitive disorders. We also observed significant racial and socioeconomic disparities in cognitive resilience. Future research should focus on methods to determine whether interventions targeting psychiatric conditions and BP control can improve cognitive outcomes in older adults. As this analysis is based on observational data, we are unable to draw definitive conclusions about causality. Despite this limitation, our study includes a large sample size, long follow‐up duration, and comprehensive assessment of clinical predictors in a real‐world primary care population.

## CONFLICT OF INTEREST STATEMENT

The authors declared no potential conflicts of interest with respect to the research, authorship, and/or publication of this article. Author disclosures are available in the supporting information.

## CONSENT STATEMENT

Consent was not necessary.

## Supporting information



Supporting Information

Supporting Information

## Data Availability

The data are not available due to privacy or ethical restrictions.
