# Supplementary material for: Predictors of cognitive resilience in the old‐old: An observational study using real‐world electronic health record data
Source: Alzheimers Dement. 2026 May 1;22(5):e71443. doi: 10.1002/alz.71443 (PMC13133552; doi:10.1002/alz.71443)
Supplement: Supplementary file 1 — Supporting Information [file ALZ-22-e71443-s002.docx]

*eTable 1. ICD-10 Diagnosis used for variable extraction in this study*

| Variable | ICD-10 codes |
| --- | --- |
| Acute Myocardial Infraction | I21.01, I21.02, I21.09, I21.11, I21.19, I21.21, I21.29, I21.3, I21.4, I21.9, I21.A1, I21.A9, I21.B, I22.0, I22.1, I22.2, I22.8, I22.9, I23.0, I23.1, I23.2, I23.3, I23.4, I23.5, I23.6, I23.7, I23.8 |
| Alcohol use disorder | F10.10, F10.120, F10.121, F10.129, F10.130, F10.131, F10.132, F10.139, F10.14, F10.150, F10.151, F10.159, F10.180, F10.181, F10.182, F10.188, F10.19, F10.20, F10.220, F10.221, F10.229, F10.230, F10.231, F10.232, F10.239, F10.24, F10.250, F10.251, F10.259, F10.26, F10.27, F10.280, F10.281, F10.282, F10.288, F10.29, F10.920, F10.921, F10.929, F10.930, F10.931, F10.932, F10.939, F10.94, F10.950, F10.951, F10.959, F10.96, F10.97, F10.980, F10.981, F10.982, F10.988, F10.99, G62.1, I42.6, K29.20, K29.21, K70.0, K70.10, K70.11, K70.2, K70.30, K70.31, K70.40, K70.41, K70.9, P04.3, Q86.0, T51.0X1A, T51.0X2A, T51.0X3A, T51.0X4A, Z71.41, Z71.42 |
| Anemia | C94.6, D46.0, D46.1, D46.20, D46.21, D46.22, D46.4, D46.9, D46.A, D46.B, D46.C, D46.Z, D47.4, D50.0, D50.1, D50.8, D50.9, D51.0, D51.1, D51.2, D51.3, D51.8, D51.9, D52.0, D52.1, D52.8, D52.9, D53.0, D53.1, D53.2, D53.8, D53.9, D55.0, D55.1, D55.2, D55.21, D55.29, D55.3, D55.8, D55.9, D56.0, D56.1, D56.2, D56.3, D56.4, D56.5, D56.8, D56.9, D57.00, D57.01, D57.02, D57.03, D57.04, D57.09, D57.1, D57.20, D57.211, D57.212, D57.213, D57.214, D57.218, D57.219, D57.3, D57.40, D57.411, D57.412, D57.413, D57.414, D57.418, D57.419, D57.42, D57.431, D57.432, D57.433, D57.434, D57.438, D57.439, D57.44, D57.451, D57.452, D57.453, D57.454, D57.458, D57.459, D57.80, D57.811, D57.812, D57.813, D57.814, D57.818, D57.819, D58.0, D58.1, D58.2, D58.8, D58.9, D59.0, D59.1, D59.10, D59.11, D59.12, D59.13, D59.19, D59.2, D59.3, D59.30, D59.31, D59.32, D59.39, D59.4, D59.5, D59.6, D59.8, D59.9, D60.0, D60.1, D60.8, D60.9, D61.01, D61.02, D61.03, D61.09, D61.1, D61.2, D61.3, D61.810, D61.811, D61.818, D61.82, D61.89, D61.9, D63.0, D63.1, D63.8, D64.0, D64.1, D64.2, D64.3, D64.4, D64.81, D64.89, D64.9, D75.81 |
| Anxiety disorders | F06.4, F40.00, F40.01, F40.02, F40.10, F40.11, F40.210, F40.218, F40.220, F40.228, F40.230, F40.231, F40.232, F40.233, F40.240, F40.241, F40.242, F40.243, F40.248, F40.290, F40.291, F40.298, F40.8, F40.9, F41.0, F41.1, F41.3, F41.8, F41.9, F42, F42.2, F42.3, F42.4, F42.8, F42.9, F43.0, F43.10, F43.11, F43.12, F44.9, F45.8, F48.8, F48.9, F93.8, F99, R45.2, R45.5, R45.6, R45.7 |
| Asthma | J45.20, J45.21, J45.22, J45.30, J45.31, J45.32, J45.40, J45.41, J45.42, J45.50, J45.51, J45.52, J45.901, J45.902, J45.909, J45.990, J45.991, J45.998 |
| Atrial Fibrillation | I48.0, I48.1, I48.11, I48.19, I48.2, I48.20, I48.21, I48.3, I48.4, I48.91 |
| Attention-Deficit/Hyperactivity Disorder | F63.0, F63.1, F63.2, F63.3, F63.81, F63.89, F63.9, F90.0, F90.1, F90.2, F90.8, F90.9, F91.0, F91.1, F91.2, F91.3, F91.8, F91.9 |
| Autism Spectrum | F84.0, F84.3, F84.5, F84.8, F84.9 |
| Benign Prostatic Hyperplasia | N40.0, N40.1, N40.2, N40.3  EXCLUSION: If any of the patients has an ICD-10 diagnosis of D29.1, they were excluded |
| Bipolar | F30.10, F30.11, F30.12, F30.13, F30.2, F30.3, F30.4, F30.8, F30.9, F31.0, F31.10, F31.11, F31.12, F31.13, F31.2, F31.30, F31.31, F31.32, F31.4, F31.5, F31.60, F31.61, F31.62, F31.63, F31.64, F31.70, F31.71, F31.72, F31.73, F31.74, F31.75, F31.76, F31.77, F31.78, F31.81, F31.89, F31.9, F33.8, F34.81, F34.89, F34.9, F39 |
| Breast Cancer | C50.011, C50.012, C50.019, C50.021, C50.022, C50.029, C50.111, C50.112, C50.119, C50.121, C50.122, C50.129, C50.211, C50.212, C50.219, C50.221, C50.222, C50.229, C50.311, C50.312, C50.319, C50.321, C50.322, C50.329, C50.411, C50.412, C50.419, C50.421, C50.422, C50.429, C50.511, C50.512, C50.519, C50.521, C50.522, C50.529, C50.611, C50.612, C50.619, C50.621, C50.622, C50.629, C50.811, C50.812, C50.819, C50.821, C50.822, C50.829, C50.911, C50.912, C50.919, C50.921, C50.922, C50.929, D05.00, D05.01, D05.02, D05.10, D05.11, D05.12, D05.80, D05.81, D05.82, D05.90, D05.91, D05.92, Z17.0, Z17.1, Z17.21, Z17.22, Z17.31, Z17.32, Z17.410, Z17.411, Z17.420, Z17.421, Z19.1, Z19.2, Z85.3, Z86.000 |
| Cataract | E08.36, E09.36, E10.36, E11.36, E13.36, H25.011, H25.012, H25.013, H25.019, H25.031, H25.032, H25.033, H25.039, H25.041, H25.042, H25.043, H25.049, H25.091, H25.092, H25.093, H25.099, H25.10, H25.11, H25.12, H25.13, H25.20, H25.21, H25.22, H25.23, H25.811, H25.812, H25.813, H25.819, H25.89, H25.9, H26.001, H26.002, H26.003, H26.009, H26.011, H26.012, H26.013, H26.019, H26.031, H26.032, H26.033, H26.039, H26.041, H26.042, H26.043, H26.049, H26.051, H26.052, H26.053, H26.059, H26.061, H26.062, H26.063, H26.069, H26.09, H26.101, H26.102, H26.103, H26.109, H26.111, H26.112, H26.113, H26.119, H26.121, H26.122, H26.123, H26.129, H26.131, H26.132, H26.133, H26.139, H26.20, H26.211, H26.212, H26.213, H26.219, H26.221, H26.222, H26.223, H26.229, H26.30, H26.31, H26.32, H26.33, H26.40, H26.411, H26.412, H26.413, H26.419, H26.491, H26.492, H26.493, H26.499, H26.8, H26.9, Q12.0 |
| Cerebral Palsy | G80.0, G80.1, G80.2, G80.3, G80.4, G80.8, G80.9 |
| Chronic Kidney Disease | A18.11, A52.75, B52.0, E08.21, E08.22, E08.29, E09.21, E09.22, E09.29, E10.21, E10.22, E10.29, E11.21, E11.22, E11.29, E13.21, E13.22, E13.29, I12.0, I12.9, I13.0, I13.10, I13.11, I13.2, K76.7, M10.30, M10.311, M10.312, M10.319, M10.321, M10.322, M10.329, M10.331, M10.332, M10.339, M10.341, M10.342, M10.349, M10.351, M10.352, M10.359, M10.361, M10.362, M10.369, M10.371, M10.372, M10.379, M10.38, M10.39, M32.14, M32.15, M35.04, M35.0A, N01.0, N01.1, N01.2, N01.3, N01.4, N01.5, N01.6, N01.7, N01.8, N01.9, N01.A, N02.0, N02.1, N02.2, N02.3, N02.4, N02.5, N02.6, N02.7, N02.8, N02.9, N02.A, N02.B1, N02.B2, N02.B3, N02.B4, N02.B5, N02.B6, N02.B9, N03.0, N03.1, N03.2, N03.3, N03.4, N03.5, N03.6, N03.7, N03.8, N03.9, N03.A, N04.0, N04.1, N04.2, N04.20, N04.21, N04.22, N04.29, N04.3, N04.4, N04.5, N04.6, N04.7, N04.8, N04.9, N04.A, N05.0, N05.1, N05.2, N05.3, N05.4, N05.5, N05.6, N05.7, N05.8, N05.9, N05.A, N06.0, N06.1, N06.2, N06.20, N06.21, N06.22, N06.29, N06.3, N06.4, N06.5, N06.6, N06.7, N06.8, N06.9, N06.A, N07.0, N07.1, N07.2, N07.3, N07.4, N07.5, N07.6, N07.7, N07.8, N07.9, N07.A, N08, N14.0, N14.1, N14.11, N14.19, N14.2, N14.3, N14.4, N15.0, N15.8, N15.9, N16, N18.1, N18.2, N18.3, N18.30, N18.31, N18.32, N18.4, N18.5, N18.6, N18.9, N25.1, N25.89, N25.9, N26.1, N26.9, N99.0, Q61.02, Q61.11, Q61.19, Q61.2, Q61.3, Q61.4, Q61.5, Q61.8 |
| Chronic Obstructive Pulmonary Disease | J 40, J41.0, J41.1, J41.8, J42, J43.0, J43.1, J43.2, J43.9, J44.0, J44.1, J44.81, J44.89, J44.9, J47.0, J47.1, J47.9, J98.2, J98.3 |
| Chronic pain | B02.22, B02.23, C34.10, C34.11, C34.12, C34.90, C34.91, C34.92, C44.01, C44.319, C44.41, C44.42, C82.00, C90.00, D04.39, D57.00, D57.03, D57.09, D57.211, D57.213, D57.218, D57.219, D57.413, D57.418, D57.419, D57.431, D57.432, D57.433, D57.438, D57.439, D57.451, D57.452, D57.453, D57.458, D57.459, D57.813, D57.818, D57.819, E10.610, E11.42, E11.44, E11.49, E11.610, E11.618, G43.011, G43.019, G43.109, G43.111, G43.119, G43.419, G43.709, G43.711, G43.719, G43.811, G43.819, G43.909, G43.911, G43.919, G43.B1, G43.E11, G43.E19, G44.001, G44.021, G44.201, G44.221, G44.301, G44.321, G44.51, G44.52, G50.0, G50.1, G56.01, G56.02, G56.03, G56.41, G56.42, G57.71, G57.72, G62.9, G89.18, G89.21, G89.28, G89.29, G89.4, G90.09, G90.50, G90.511, G90.512, G90.513, G90.519, G90.521, G90.522, G90.529, I20.0, I25.10, I25.110, I25.118, I25.119, I25.5, I25.84, I42.9, I70.213, I70.221, I70.222, I70.223, I70.228, I70.229, I70.321, I70.322, I70.521, I70.522, I70.523, I73.00, I73.9, K74.60, K76.0, K76.89, M05.79, M06.00, M06.0A, M06.9, M10.071, M10.072, M10.079, M10.9, M13.851, M13.852, M13.859, M13.871, M13.872, M13.879, M15.0, M15.9, M16.0, M16.10, M16.11, M16.12, M17.0, M17.10, M17.11, M17.12, M17.30, M17.31, M17.32, M17.9, M18.10, M18.11, M18.12, M19.011, M19.012, M19.019, M19.031, M19.032, M19.039, M19.041, M19.042, M19.049, M19.071, M19.072, M19.079, M19.90, M23.321, M23.322, M23.329, M25.361, M25.362, M25.369, M25.421, M25.422, M25.429, M25.461, M25.462, M25.469, M25.471, M25.472, M25.473, M25.50, M25.511, M25.512, M25.519, M25.521, M25.522, M25.529, M25.531, M25.532, M25.539, M25.541, M25.542, M25.549, M25.551, M25.552, M25.559, M25.561, M25.562, M25.569, M25.571, M25.572, M25.579, M25.59, M25.60, M25.611, M25.612, M25.619, M25.661, M25.662, M25.669, M25.69, M25.78, M32.9, M41.9, M43.16, M43.6, M45.0, M45.AB, M46.1, M47.26, M47.812, M47.814, M47.816, M47.817, M47.896, M48.00, M48.02, M48.06, M48.061, M48.062, M48.07, M48.56XA, M48.56XD, M48.56XG, M48.56XS, M50.32, M50.321, M50.322, M50.323, M51.16, M51.26, M51.36, M51.37, M53.2X7, M53.81, M54.12, M54.16, M54.17, M54.2, M54.30, M54.31, M54.32, M54.40, M54.41, M54.42, M54.5, M54.50, M54.51, M54.59, M54.6, M54.9, M62.49, M70.60, M70.61, M70.62, M71.20, M71.21, M71.22, M71.9, M75.100, M75.101, M75.102, M75.120, M75.121, M75.122, M75.50, M75.51, M75.52, M79.1, M79.10, M79.11, M79.12, M79.18, M79.601, M79.602, M79.604, M79.605, M79.609, M79.621, M79.622, M79.641, M79.642, M79.643, M79.644, M79.645, M79.646, M79.661, M79.662, M79.671, M79.672, M79.673, M79.674, M79.675, M79.676, M79.7, M79.89, M80.00XA, M80.00XD, M80.00XG, M80.00XK, M80.00XP, M80.00XS, M80.08XA, M80.08XD, M80.08XG, M80.08XK, M80.08XP, M80.08XS, M80.0AXA, M80.0AXD, M80.0AXG, M80.0AXK, M80.0AXP, M80.0AXS, M87.9, M96.1, M99.04, M99.05, N18.3, N18.30, N18.31, N18.32, N18.9, N23, N41.0, N41.1, R07.0, R07.2, R07.81, R07.89, R07.9, R10.11, R10.12, R10.13, R10.2, R10.30, R10.31, R10.32, R10.84, R10.9, R30.0, R30.9, R39.82, R51, R51.0, R51.9, R52, S22.41XA, S22.41XB, S22.41XD, S22.41XG, S22.41XK, S22.41XS, S22.42XA, S22.42XB, S22.42XD, S22.42XG, S22.42XK, S22.42XS, S22.49XA, S22.49XB, S22.49XD, S22.49XG, S22.49XK, S22.49XS, S32.010A, S32.010B, S32.010D, S32.010G, S32.010K, S32.010S, S32.020A, S32.020B, S32.020D, S32.020G, S32.020K, S32.020S, S32.030A, S32.030B, S32.030D, S32.030G, S32.030K, S32.030S, S32.040A, S32.040B, S32.040D, S32.040G, S32.040K, S32.040S, S32.050A, S32.050B, S32.050D, S32.050G, S32.050K, S32.050S, S32.10XA, S32.10XB, S32.10XD, S32.10XG, S32.10XK, S32.10XS, S32.591A, S32.591B, S32.591D, S32.591G, S32.591K, S32.591S, S32.592A, S32.592B, S32.592D, S32.592G, S32.592K, S32.592S, S32.599A, S32.599B, S32.599D, S32.599G, S32.599K, S32.599S, S32.810A, S32.810B, S32.810D, S32.810G, S32.810K, S32.810S, S32.82XA, S32.82XB, S32.82XD, S32.82XG, S32.82XK, S32.82XS, S32.89XA, S32.89XB, S32.89XD, S32.89XG, S32.89XK, S32.89XS |
| Colorectal Cancer | C18.0, C18.1, C18.2, C18.3, C18.4, C18.5, C18.6, C18.7, C18.8, C18.9, C19, C20, C49.A4, C49.A5, D01.0, D01.1, D01.2, Z85.030, Z85.038, Z85.040, Z85.048 |
| Cystic fibrosis | D81.810, D84.1, E00.0, E00.1, E00.2, E00.9, E03.0, E03.1, E25.0, E25.8, E25.9, E56.9, E70.0, E70.1, E70.20, E70.21, E70.29, E70.30, E70.310, E70.311, E70.318, E70.319, E70.320, E70.321, E70.328, E70.329, E70.330, E70.331, E70.338, E70.339, E70.39, E70.5, E70.8, E70.81, E70.89, E70.9, E71.0, E71.110, E71.111, E71.118, E71.19, E71.2, E71.310, E71.311, E71.312, E71.313, E71.314, E71.318, E71.32, E71.41, E72.10, E72.11, E72.12, E72.19, E72.20, E72.21, E72.22, E72.23, E72.29, E72.3, E72.4, E72.50, E72.51, E72.59, E72.8, E74.20, E74.21, E74.29, E74.810, E74.818, E74.819, E74.820, E74.829, E74.89, E84.0, E84.11, E84.19, E84.8, E84.9, E88.82 |
| Depression/ Depressive Mood Disorders | F06.31, F06.32, F31.0, F31.10, F31.11, F31.12, F31.13, F31.2, F31.30, F31.31, F31.32, F31.4, F31.5, F31.60, F31.61, F31.62, F31.63, F31.64, F31.71, F31.73, F31.75, F31.76, F31.77, F31.78, F31.81, F31.89, F31.9, F32.0, F32.1, F32.2, F32.3, F32.4, F32.5, F32.8, F32.89, F32.9, F32.A, F33.0, F33.1, F33.2, F33.3, F33.40, F33.41, F33.42, F33.8, F33.9, F34.0, F34.1, F43.21, F43.23 |
| Diabetes | E08.00, E08.01, E08.10, E08.11, E08.21, E08.22, E08.29, E08.311, E08.319, E08.321, E08.3211, E08.3212, E08.3213, E08.3219, E08.329, E08.3291, E08.3292, E08.3293, E08.3299, E08.331, E08.3311, E08.3312, E08.3313, E08.3319, E08.339, E08.3391, E08.3392, E08.3393, E08.3399, E08.341, E08.3411, E08.3412, E08.3413, E08.3419, E08.349, E08.3491, E08.3492, E08.3493, E08.3499, E08.351, E08.3511, E08.3512, E08.3513, E08.3519, E08.3521, E08.3522, E08.3523, E08.3529, E08.3531, E08.3532, E08.3533, E08.3539, E08.3541, E08.3542, E08.3543, E08.3549, E08.3551, E08.3552, E08.3553, E08.3559, E08.359, E08.3591, E08.3592, E08.3593, E08.3599, E08.36, E08.37X1, E08.37X2, E08.37X3, E08.37X9, E08.39, E08.40, E08.41, E08.42, E08.43, E08.44, E08.49, E08.51, E08.52, E08.59, E08.610, E08.618, E08.620, E08.621, E08.622, E08.628, E08.630, E08.638, E08.641, E08.649, E08.65, E08.69, E08.8, E08.9, E09.00, E09.01, E09.10, E09.11, E09.21, E09.22, E09.29, E09.311, E09.319, E09.321, E09.3211, E09.3212, E09.3213, E09.3219, E09.329, E09.3291, E09.3292, E09.3293, E09.3299, E09.331, E09.3311, E09.3312, E09.3313, E09.3319, E09.339, E09.3391, E09.3392, E09.3393, E09.3399, E09.341, E09.3411, E09.3412, E09.3413, E09.3419, E09.349, E09.3491, E09.3492, E09.3493, E09.3499, E09.351, E09.3511, E09.3512, E09.3513, E09.3519, E09.3521, E09.3522, E09.3523, E09.3529, E09.3531, E09.3532, E09.3533, E09.3539, E09.3541, E09.3542, E09.3543, E09.3549, E09.3551, E09.3552, E09.3553, E09.3559, E09.359, E09.3591, E09.3592, E09.3593, E09.3599, E09.36, E09.37X1, E09.37X2, E09.37X3, E09.37X9, E09.39, E09.40, E09.41, E09.42, E09.43, E09.44, E09.49, E09.51, E09.52, E09.59, E09.610, E09.618, E09.620, E09.621, E09.622, E09.628, E09.630, E09.638, E09.641, E09.649, E09.65, E09.69, E09.8, E09.9, E10.10, E10.11, E10.21, E10.22, E10.29, E10.311, E10.319, E10.321, E10.3211, E10.3212, E10.3213, E10.3219, E10.329, E10.3291, E10.3292, E10.3293, E10.3299, E10.331, E10.3311, E10.3312, E10.3313, E10.3319, E10.339, E10.3391, E10.3392, E10.3393, E10.3399, E10.341, E10.3411, E10.3412, E10.3413, E10.3419, E10.349, E10.3491, E10.3492, E10.3493, E10.3499, E10.351, E10.3511, E10.3512, E10.3513, E10.3519, E10.3521, E10.3522, E10.3523, E10.3529, E10.3531, E10.3532, E10.3533, E10.3539, E10.3541, E10.3542, E10.3543, E10.3549, E10.3551, E10.3552, E10.3553, E10.3559, E10.359, E10.3591, E10.3592, E10.3593, E10.3599, E10.36, E10.37X1, E10.37X2, E10.37X3, E10.37X9, E10.39, E10.40, E10.41, E10.42, E10.43, E10.44, E10.49, E10.51, E10.52, E10.59, E10.610, E10.618, E10.620, E10.621, E10.622, E10.628, E10.630, E10.638, E10.641, E10.649, E10.65, E10.69, E10.8, E10.9, E10.A0, E10.A1, E10.A2, E11.00, E11.01, E11.10, E11.11, E11.21, E11.22, E11.29, E11.311, E11.319, E11.321, E11.3211, E11.3212, E11.3213, E11.3219, E11.329, E11.3291, E11.3292, E11.3293, E11.3299, E11.331, E11.3311, E11.3312, E11.3313, E11.3319, E11.339, E11.3391, E11.3392, E11.3393, E11.3399, E11.341, E11.3411, E11.3412, E11.3413, E11.3419, E11.349, E11.3491, E11.3492, E11.3493, E11.3499, E11.351, E11.3511, E11.3512, E11.3513, E11.3519, E11.3521, E11.3522, E11.3523, E11.3529, E11.3531, E11.3532, E11.3533, E11.3539, E11.3541, E11.3542, E11.3543, E11.3549, E11.3551, E11.3552, E11.3553, E11.3559, E11.359, E11.3591, E11.3592, E11.3593, E11.3599, E11.36, E11.37X1, E11.37X2, E11.37X3, E11.37X9, E11.39, E11.40, E11.41, E11.42, E11.43, E11.44, E11.49, E11.51, E11.52, E11.59, E11.610, E11.618, E11.620, E11.621, E11.622, E11.628, E11.630, E11.638, E11.641, E11.649, E11.65, E11.69, E11.8, E11.9, E13.00, E13.01, E13.10, E13.11, E13.21, E13.22, E13.29, E13.311, E13.319, E13.321, E13.3211, E13.3212, E13.3213, E13.3219, E13.329, E13.3291, E13.3292, E13.3293, E13.3299, E13.331, E13.3311, E13.3312, E13.3313, E13.3319, E13.339, E13.3391, E13.3392, E13.3393, E13.3399, E13.341, E13.3411, E13.3412, E13.3413, E13.3419, E13.349, E13.3491, E13.3492, E13.3493, E13.3499, E13.351, E13.3511, E13.3512, E13.3513, E13.3519, E13.3521, E13.3522, E13.3523, E13.3529, E13.3531, E13.3532, E13.3533, E13.3539, E13.3541, E13.3542, E13.3543, E13.3549, E13.3551, E13.3552, E13.3553, E13.3559, E13.359, E13.3591, E13.3592, E13.3593, E13.3599, E13.36, E13.39, E13.40, E13.41, E13.42, E13.43, E13.44, E13.49, E13.51, E13.52, E13.59, E13.610, E13.618, E13.620, E13.621, E13.622, E13.628, E13.630, E13.638, E13.641, E13.649, E13.65, E13.69, E13.8, E13.9 |
| Drug use disorders | F11.10, F11.120, F11.121, F11.122, F11.129, F11.13, F11.14, F11.150, F11.151, F11.159, F11.181, F11.182, F11.188, F11.19, F11.20, F11.220, F11.221, F11.222, F11.229, F11.23, F11.24, F11.250, F11.251, F11.259, F11.281, F11.282, F11.288, F11.29, F11.90, F11.920, F11.921, F11.922, F11.929, F11.93, F11.94, F11.950, F11.951, F11.959, F11.981, F11.982, F11.988, F11.99, F12.10, F12.120, F12.121, F12.122, F12.129, F12.13, F12.150, F12.151, F12.159, F12.180, F12.188, F12.19, F12.20, F12.220, F12.221, F12.222, F12.229, F12.250, F12.251, F12.259, F12.280, F12.288, F12.29, F12.90, F12.920, F12.921, F12.922, F12.929, F12.950, F12.951, F12.959, F12.980, F12.988, F12.99, F13.10, F13.120, F13.121, F13.129, F13.130, F13.131, F13.132, F13.139, F13.14, F13.150, F13.151, F13.159, F13.180, F13.181, F13.182, F13.188, F13.19, F13.20, F13.220, F13.221, F13.229, F13.230, F13.231, F13.232, F13.239, F13.24, F13.250, F13.251, F13.259, F13.26, F13.27, F13.280, F13.281, F13.282, F13.288, F13.29, F13.90, F13.920, F13.921, F13.929, F13.930, F13.931, F13.932, F13.939, F13.94, F13.950, F13.951, F13.959, F13.96, F13.97, F13.980, F13.981, F13.982, F13.988, F13.99, F14.10, F14.120, F14.121, F14.122, F14.129, F14.13, F14.14, F14.150, F14.151, F14.159, F14.180, F14.181, F14.182, F14.188, F14.19, F14.20, F14.220, F14.221, F14.222, F14.229, F14.23, F14.24, F14.250, F14.251, F14.259, F14.280, F14.281, F14.282, F14.288, F14.29, F14.90, F14.920, F14.921, F14.922, F14.929, F14.93, F14.94, F14.950, F14.951, F14.959, F14.980, F14.981, F14.982, F14.988, F14.99, F15.10, F15.120, F15.121, F15.122, F15.129, F15.13, F15.14, F15.150, F15.151, F15.159, F15.180, F15.181, F15.182, F15.188, F15.19, F15.20, F15.220, F15.221, F15.222, F15.229, F15.23, F15.24, F15.250, F15.251, F15.259, F15.280, F15.281, F15.282, F15.288, F15.29, F15.90, F15.920, F15.921, F15.922, F15.929, F15.93, F15.94, F15.950, F15.951, F15.959, F15.980, F15.981, F15.982, F15.988, F15.99, F16.10, F16.120, F16.121, F16.122, F16.129, F16.14, F16.150, F16.151, F16.159, F16.180, F16.183, F16.188, F16.19, F16.20, F16.220, F16.221, F16.229, F16.24, F16.250, F16.251, F16.259, F16.280, F16.283, F16.288, F16.29, F16.90, F16.920, F16.921, F16.929, F16.94, F16.950, F16.951, F16.959, F16.980, F16.983, F16.988, F16.99, F17.203, F17.208, F17.209, F17.213, F17.218, F17.219, F17.223, F17.228, F17.229, F17.293, F17.298, F17.299, F18.10, F18.120, F18.121, F18.129, F18.14, F18.150, F18.151, F18.159, F18.17, F18.180, F18.188, F18.19, F18.20, F18.220, F18.221, F18.229, F18.24, F18.250, F18.251, F18.259, F18.27, F18.280, F18.288, F18.29, F18.90, F18.920, F18.921, F18.929, F18.94, F18.950, F18.951, F18.959, F18.97, F18.980, F18.988, F18.99, F19.10, F19.120, F19.121, F19.122, F19.129, F19.130, F19.131, F19.132, F19.139, F19.14, F19.150, F19.151, F19.159, F19.16, F19.17, F19.180, F19.181, F19.182, F19.188, F19.19, F19.20, F19.220, F19.221, F19.222, F19.229, F19.230, F19.231, F19.232, F19.239, F19.24, F19.250, F19.251, F19.259, F19.26, F19.27, F19.280, F19.281, F19.282, F19.288, F19.29, F19.90, F19.920, F19.921, F19.922, F19.929, F19.930, F19.931, F19.932, F19.939, F19.94, F19.950, F19.951, F19.959, F19.96, F19.97, F19.980, F19.981, F19.982, F19.988, F19.99, F55.0, F55.1, F55.2, F55.3, F55.4, F55.8, O35.5XX0, O35.5XX1, O35.5XX2, O35.5XX3, O35.5XX4, O35.5XX5, O35.5XX9, O99.320, O99.321, O99.322, O99.323, O99.324, O99.325, P04.41, P04.49, P96.1, P96.2, T40.0X1A, T40.0X2A, T40.0X3A, T40.0X4A, T40.0X5A, T40.0X5S, T40.1X1A, T40.1X2A, T40.1X3A, T40.1X4A, T40.2X1A, T40.2X2A, T40.2X3A, T40.2X4A, T40.3X1A, T40.3X2A, T40.3X3A, T40.3X4A, T40.3X5A, T40.3X5S, T40.411A, T40.412A, T40.413A, T40.414A, T40.415A, T40.421A, T40.422A, T40.423A, T40.424A, T40.425A, T40.491A, T40.492A, T40.493A, T40.494A, T40.495A, T40.4X1A, T40.4X2A, T40.4X3A, T40.4X4A, T40.601A, T40.602A, T40.603A, T40.604A, T40.691A, T40.692A, T40.693A, T40.694A, T40.711A, T40.721A, T40.7X1A, T40.8X1A, T40.901A, T40.991A, Z71.41, Z71.42, Z71.51, Z71.52, Z71.6 |
| Endometrial Cancer | C54.0, C54.1, C54.2, C54.3, C54.8, C54.9, D07.0, Z85.42 |
| Epilepsy | G40.001, G40.009, G40.011, G40.019, G40.101, G40.109, G40.111, G40.119, G40.201, G40.209, G40.211, G40.219, G40.301, G40.309, G40.311, G40.319, G40.401, G40.409, G40.411, G40.419, G40.42, G40.501, G40.509, G40.801, G40.802, G40.803, G40.804, G40.811, G40.812, G40.813, G40.814, G40.821, G40.822, G40.823, G40.824, G40.833, G40.834, G40.841, G40.842, G40.843, G40.844, G40.89, G40.901, G40.909, G40.911, G40.919, G40.A01, G40.A09, G40.A11, G40.A19, G40.B01, G40.B09, G40.B11, G40.B19, G40.C01, G40.C09, G40.C11, G40.C19, G93.45 |
| Glaucoma | H40.011, H40.012, H40.013, H40.019, H40.021, H40.022, H40.023, H40.029, H40.041, H40.042, H40.043, H40.049, H40.051, H40.052, H40.053, H40.059, H40.10X0, H40.10X1, H40.10X2, H40.10X3, H40.10X4, H40.1110, H40.1111, H40.1112, H40.1113, H40.1114, H40.1120, H40.1121, H40.1122, H40.1123, H40.1124, H40.1130, H40.1131, H40.1132, H40.1133, H40.1134, H40.1190, H40.1191, H40.1192, H40.1193, H40.1194, H40.11X0, H40.11X1, H40.11X2, H40.11X3, H40.11X4, H40.1210, H40.1211, H40.1212, H40.1213, H40.1214, H40.1220, H40.1221, H40.1222, H40.1223, H40.1224, H40.1230, H40.1231, H40.1232, H40.1233, H40.1234, H40.1290, H40.1291, H40.1292, H40.1293, H40.1294, H40.1310, H40.1311, H40.1312, H40.1313, H40.1314, H40.1320, H40.1321, H40.1322, H40.1323, H40.1324, H40.1330, H40.1331, H40.1332, H40.1333, H40.1334, H40.1390, H40.1391, H40.1392, H40.1393, H40.1394, H40.1410, H40.1411, H40.1412, H40.1413, H40.1414, H40.1420, H40.1421, H40.1422, H40.1423, H40.1424, H40.1430, H40.1431, H40.1432, H40.1433, H40.1434, H40.1490, H40.1491, H40.1492, H40.1493, H40.1494, H40.151, H40.152, H40.153, H40.159, H40.20X0, H40.20X1, H40.20X2, H40.20X3, H40.20X4, H40.211, H40.212, H40.213, H40.219, H40.2210, H40.2211, H40.2212, H40.2213, H40.2214, H40.2220, H40.2221, H40.2222, H40.2223, H40.2224, H40.2230, H40.2231, H40.2232, H40.2233, H40.2234, H40.2290, H40.2291, H40.2292, H40.2293, H40.2294, H40.231, H40.232, H40.233, H40.239, H40.241, H40.242, H40.243, H40.249, H40.30X0, H40.30X1, H40.30X2, H40.30X3, H40.30X4, H40.31X0, H40.31X1, H40.31X2, H40.31X3, H40.31X4, H40.32X0, H40.32X1, H40.32X2, H40.32X3, H40.32X4, H40.33X0, H40.33X1, H40.33X2, H40.33X3, H40.33X4, H40.40X0, H40.40X1, H40.40X2, H40.40X3, H40.40X4, H40.41X0, H40.41X1, H40.41X2, H40.41X3, H40.41X4, H40.42X0, H40.42X1, H40.42X2, H40.42X3, H40.42X4, H40.43X0, H40.43X1, H40.43X2, H40.43X3, H40.43X4, H40.50X0, H40.50X1, H40.50X2, H40.50X3, H40.50X4, H40.51X0, H40.51X1, H40.51X2, H40.51X3, H40.51X4, H40.52X0, H40.52X1, H40.52X2, H40.52X3, H40.52X4, H40.53X0, H40.53X1, H40.53X2, H40.53X3, H40.53X4, H40.60X0, H40.60X1, H40.60X2, H40.60X3, H40.60X4, H40.61X0, H40.61X1, H40.61X2, H40.61X3, H40.61X4, H40.62X0, H40.62X1, H40.62X2, H40.62X3, H40.62X4, H40.63X0, H40.63X1, H40.63X2, H40.63X3, H40.63X4, H40.811, H40.812, H40.813, H40.819, H40.821, H40.822, H40.823, H40.829, H40.831, H40.832, H40.833, H40.839, H40.89, H40.9, H42, H44.511, H44.512, H44.513, H44.519, H47.231, H47.232, H47.233, H47.239, Q15.0 |
| Heart Failure | I09.81, I11.0, I13.0, I13.2, I42.0, I42.5, I42.6, I42.7, I42.8, I43, I50.1, I50.20, I50.21, I50.22, I50.23, I50.30, I50.31, I50.32, I50.33, I50.40, I50.41, I50.42, I50.43, I50.810, I50.811, I50.812, I50.813, I50.814, I50.82, I50.83, I50.84, I50.89, I50.9, P29.0 |
| Hyperlipidemia | E78.0, E78.00, E78.01, E78.1, E78.2, E78.3, E78.4, E78.41, E78.49, E78.5 |
| Hypertension | H35.031, H35.032, H35.033, H35.039, I10, I11.0, I11.9, I12.0, I12.9, I13.0, I13.10, I13.11, I13.2, I15.0, I15.1, I15.2, I15.8, I15.9, I1A.0, I67.4, N26.2 |
| Hypothyroidism | E00.0, E00.1, E00.2, E00.9, E01.8, E02, E03.0, E03.1, E03.2, E03.3, E03.4, E03.8, E03.9, E89.0 |
| Intellectual Disabilities and Related Conditions | E78.71, E78.72, F70, F71, F72, F73, F78, F78.A1, F78.A9, F79, P04.3, Q86.0, Q87.1, Q87.11, Q87.19, Q87.2, Q87.3, Q87.5, Q87.81, Q87.83, Q87.84, Q87.85, Q87.86, Q87.89, Q89.7, Q89.8, Q90.0, Q90.1, Q90.2, Q90.9, Q91.0, Q91.1, Q91.2, Q91.3, Q91.4, Q91.5, Q91.6, Q91.7, Q92.0, Q92.1, Q92.2, Q92.5, Q92.61, Q92.62, Q92.7, Q92.8, Q92.9, Q93.0, Q93.1, Q93.2, Q93.3, Q93.4, Q93.5, Q93.51, Q93.52, Q93.59, Q93.7, Q93.81, Q93.88, Q93.89, Q93.9, Q95.2, Q95.3, Q99.2 |
| Ischemic heart disease | I20.0, I20.1, I20.2, I20.8, I20.81, I20.89, I24.0, I24.1, I24.8, I24.81, I24.89, I25.10, I25.110, I25.111, I25.112, I25.118, I25.119, I25.3, I25.41, I25.42, I25.5, I25.6, I25.700, I25.701, I25.702, I25.708, I25.710, I25.711, I25.712, I25.718, I25.719, I25.720, I25.721, I25.722, I25.728, I25.729, I25.730, I25.731, I25.732, I25.738, I25.739, I25.750, I25.751, I25.752, I25.758, I25.759, I25.760, I25.761, I25.762, I25.768, I25.769, I25.790, I25.791, I25.792, I25.798, I25.799, I25.810, I25.811, I25.812, I25.82, I25.83, I25.84, I25.85, I25.89, I25.9 |
| Learning disabilities | F80.0, F80.1, F80.2, F80.4, F80.81, F80.82, F80.89, F80.9, F81.0, F81.2, F81.81, F81.89, F81.9, F82, H93.25, R48.0 |
| Lung Cancer | C34.00, C34.01, C34.02, C34.10, C34.11, C34.12, C34.2, C34.30, C34.31, C34.32, C34.80, C34.81, C34.82, C34.90, C34.91, C34.92, D02.20, D02.21, D02.22, Z85.110, Z85.118 |
| Mobility impairments | G04.1, G11.4, G81.00, G81.01, G81.02, G81.03, G81.04, G81.10, G81.11, G81.12, G81.13, G81.14, G81.90, G81.91, G81.92, G81.93, G81.94, G82.20, G82.21, G82.22, G82.50, G82.51, G82.52, G82.53, G82.54, G83.0, G83.10, G83.11, G83.12, G83.13, G83.14, G83.20, G83.21, G83.22, G83.23, G83.24, G83.30, G83.31, G83.32, G83.33, G83.34, G83.4, G83.5, G83.81, G83.82, G83.83, G83.84, G83.89, G83.9, I69.031, I69.032, I69.033, I69.034, I69.039, I69.041, I69.042, I69.043, I69.044, I69.049, I69.051, I69.052, I69.053, I69.054, I69.059, I69.061, I69.062, I69.063, I69.064, I69.065, I69.069, I69.131, I69.132, I69.133, I69.134, I69.139, I69.141, I69.142, I69.143, I69.144, I69.149, I69.151, I69.152, I69.153, I69.154, I69.159, I69.161, I69.162, I69.163, I69.164, I69.165, I69.169, I69.231, I69.232, I69.233, I69.234, I69.239, I69.241, I69.242, I69.243, I69.244, I69.249, I69.251, I69.252, I69.253, I69.254, I69.259, I69.261, I69.262, I69.263, I69.264, I69.265, I69.269, I69.331, I69.332, I69.333, I69.334, I69.339, I69.341, I69.342, I69.343, I69.344, I69.349, I69.351, I69.352, I69.353, I69.354, I69.359, I69.361, I69.362, I69.363, I69.364, I69.365, I69.369, I69.831, I69.832, I69.833, I69.834, I69.839, I69.841, I69.842, I69.843, I69.844, I69.849, I69.851, I69.852, I69.853, I69.854, I69.859, I69.861, I69.862, I69.863, I69.864, I69.865, I69.869, I69.931, I69.932, I69.933, I69.934, I69.939, I69.941, I69.942, I69.943, I69.944, I69.949, I69.951, I69.952, I69.953, I69.954, I69.959, I69.961, I69.962, I69.963, I69.964, I69.965, I69.969 |
| Obesity | E66.01, E66.09, E66.1, E66.2, E66.8, E66.811, E66.812, E66.813, E66.89, E66.9, E88.82, Z68.30, Z68.31, Z68.32, Z68.33, Z68.34, Z68.35, Z68.36, Z68.37, Z68.38, Z68.39, Z68.41, Z68.42, Z68.43, Z68.44, Z68.45, Z68.55, Z68.56 |
| Opioid use disorder | F11.10, F11.120, F11.121, F11.122, F11.129, F11.13, F11.14, F11.150, F11.151, F11.159, F11.181, F11.182, F11.188, F11.19, F11.20, F11.220, F11.221, F11.222, F11.229, F11.23, F11.24, F11.250, F11.251, F11.259, F11.281, F11.282, F11.288, F11.29, F11.90, F11.920, F11.921, F11.922, F11.929, F11.93, F11.94, F11.950, F11.951, F11.959, F11.981, F11.982, F11.988, F11.99, T40.0X1A, T40.0X2A, T40.0X3A, T40.0X4A, T40.1X1A, T40.1X2A, T40.1X3A, T40.1X4A, T40.2X1A, T40.2X2A, T40.2X3A, T40.2X4A, T40.3X1A, T40.3X2A, T40.3X3A, T40.3X4A, T40.3X5A, T40.4X1A, T40.4X2A, T40.4X3A, T40.4X4A, T40.411A, T40.412A, T40.413A, T40.414A, T40.415A, T40.421A, T40.422A, T40.423A, T40.424A, T40.425A, T40.491A, T40.492A, T40.493A, T40.494A, T40.495A, T40.601A, T40.602A, T40.603A, T40.604A, T40.691A, T40.692A, T40.693A, T40.694A |
| Osteoporosis | M80.00XA, M80.011A, M80.012A, M80.019A, M80.021A, M80.022A, M80.029A, M80.031A, M80.032A, M80.039A, M80.041A, M80.042A, M80.049A, M80.051A, M80.052A, M80.059A, M80.061A, M80.062A, M80.069A, M80.071A, M80.072A, M80.079A, M80.08XA, M80.0AXA, M80.0B1A, M80.0B2A, M80.0B9A, M80.80XA, M80.811A, M80.812A, M80.819A, M80.821A, M80.822A, M80.829A, M80.831A, M80.832A, M80.839A, M80.841A, M80.842A, M80.849A, M80.851A, M80.852A, M80.859A, M80.861A, M80.862A, M80.869A, M80.871A, M80.872A, M80.879A, M80.88XA, M80.8AXA, M80.8B1A, M80.8B2A, M80.8B9A, M81.0, M81.6, M81.8 |
| Parkinson’s | G20, G20.A1, G20.A2, G20.B1, G20.B2, G20.C, G21.11, G21.19, G21.3, G21.4, G21.8, G21.9, G31.83 |
| Personality disorders | F21, F34.0, F34.1, F60.0, F60.1, F60.2, F60.3, F60.4, F60.5, F60.6, F60.7, F60.81, F60.89, F60.9, F68.10, F68.11, F68.12, F68.13, F69 |
| Pneumonia | A01.03, A02.22, A06.5, A20.2, A21.2, A22.1, A31.0, A37.01, A37.11, A37.81, A37.91, A40.3, A42.0, A43.0, A48.1, A50.04, A54.84, B01.2, B05.2, B06.81, B37.1, B38.0, B38.2, B39.0, B39.2, B40.0, B40.2, B41.0, B58.3, B59, B66.4, B67.1, B77.81, B95.3, B96.0, B96.1, J09.X1, J10.00, J10.01, J10.08, J11.00, J11.08, J12.0, J12.1, J12.2, J12.3, J12.81, J12.82, J12.89, J12.9, J13, J14, J15.0, J15.1, J15.20, J15.211, J15.212, J15.29, J15.3, J15.4, J15.5, J15.6, J15.61, J15.69, J15.7, J15.8, J15.9, J16.0, J16.8, J17, J18.0, J18.1, J18.2, J18.8, J18.9, J20.0, J84.111, J84.116, J84.117, J84.178, J84.2, J85.1, J95.851, P23.0, P23.1, P23.2, P23.3, P23.4, P23.5, P23.6, P23.8, P23.9, Z87.01 |
| Post traumatic stress disorder | F43.10, F43.11, F43.12 |
| Prostate Cancer | C61, D07.5, Z85.46 |
| Stroke/ Transient Ischemic Attack | G45.0, G45.1, G45.2, G45.3, G45.8, G45.9, G46.0, G46.1, G46.2, G46.3, G46.4, G46.5, G46.6, G46.7, G46.8, G97.31, G97.32, I60.00, I60.01, I60.02, I60.10, I60.11, I60.12, I60.2, I60.20, I60.21, I60.22, I60.30, I60.31, I60.32, I60.4, I60.50, I60.51, I60.52, I60.6, I60.7, I60.8, I60.9, I61.0, I61.1, I61.2, I61.3, I61.4, I61.5, I61.6, I61.8, I61.9, I62.00, I62.01, I62.02, I62.9, I63.00, I63.011, I63.012, I63.013, I63.019, I63.02, I63.031, I63.032, I63.033, I63.039, I63.09, I63.10, I63.111, I63.112, I63.113, I63.119, I63.12, I63.131, I63.132, I63.133, I63.139, I63.19, I63.20, I63.211, I63.212, I63.213, I63.219, I63.22, I63.231, I63.232, I63.233, I63.239, I63.29, I63.30, I63.311, I63.312, I63.313, I63.319, I63.321, I63.322, I63.323, I63.329, I63.331, I63.332, I63.333, I63.339, I63.341, I63.342, I63.343, I63.349, I63.39, I63.40, I63.411, I63.412, I63.413, I63.419, I63.421, I63.422, I63.423, I63.429, I63.431, I63.432, I63.433, I63.439, I63.441, I63.442, I63.443, I63.449, I63.49, I63.50, I63.511, I63.512, I63.513, I63.519, I63.521, I63.522, I63.523, I63.529, I63.531, I63.532, I63.533, I63.539, I63.541, I63.542, I63.543, I63.549, I63.59, I63.6, I63.8, I63.81, I63.89, I63.9, I67.841, I67.848, I67.89, I97.810, I97.811, I97.820, I97.821 (any DX on the claim)  EXCLUSION: If any of the patients had any of the following codes in any DX position they were excluded: S06.340A, S06.341A, S06.342A, S06.343A, S06.344A, S06.345A, S06.346A, S06.347A, S06.348A, S06.34AA, S06.349A, S06.350A, S06.351A, S06.352A, S06.353A, S06.354A, S06.355A, S06.356A, S06.357A, S06.358A, S06.35AA, S06.359A, S06.360A, S06.361A, S06.362A, S06.363A, S06.364A, S06.365A, S06.366A, S06.367A, S06.368A, S06.36AA, S06.369A, S06.370A, S06.371A, S06.372A, S06.373A, S06.374A, S06.375A, S06.376A, S06.377A, S06.378A, S06.37AA, S06.379A, S06.380A, S06.381A, S06.382A, S06.383A, S06.384A, S06.385A, S06.386A, S06.387A, S06.388A, S06.38AA, S06.389A, S06.5X0A, S06.5X1A, S06.5X2A, S06.5X3A, S06.5X4A, S06.5X5A, S06.5X6A, S06.5X7A, S06.5X8A, S06.5XAA, S06.5X9A, S06.6X0A, S06.6X1A, S06.6X2A, S06.6X3A, S06.6X4A, S06.6X5A, S06.6X6A, S06.6X7A, S06.6X8A, S06.6XAA, S06.6X9A, S06.810A, S06.811A, S06.812A, S06.813A, S06.814A, S06.815A, S06.816A, S06.817A, S06.818A, S06.81AA, S06.819A, S06.820A, S06.821A, S06.822A, S06.823A, S06.824A, S06.825A, S06.826A, S06.827A, S06.828A, S06.82AA, S06.829A, S06.890A, S06.891A, S06.892A, S06.893A, S06.894A, S06.895A, S06.896A, S06.897A, S06.898A, S06.89AA, S06.899A, S06.9X0A, S06.9X1A, S06.9X2A, S06.9X3A, S06.9X4A, S06.9X5A, S06.9X6A, S06.9X7A, S06.9X8A, S06.9XAA, S06.9X9A, S06.A0XA, S06.A1XA |
| Schizophrenia | F20.0, F20.1, F20.2, F20.3, F20.5, F20.81, F20.89, F20.9, F25.0, F25.1, F25.8, F25.9 |
| Sensory hearing impairment | H90.3, H90.41, H90.42, H90.5, H90.6, H90.71, H90.72, H90.8, H90.A21, H90.A22, H90.A31, H90.A32, H91.01, H91.02, H91.03, H91.09, H91.3, H91.8X1, H91.8X2, H91.8X3, H91.8X9, H91.90, H91.91, H91.92, H91.93 |
| Sensory visual impairment | H54.0, H54.0X33, H54.0X34, H54.0X35, H54.0X43, H54.0X44, H54.0X45, H54.0X53, H54.0X54, H54.0X55, H54.10, H54.11, H54.1131, H54.1132, H54.1141, H54.1142, H54.1151, H54.1152, H54.12, H54.1213, H54.1214, H54.1215, H54.1223, H54.1224, H54.1225, H54.2, H54.2X11, H54.2X12, H54.2X21, H54.2X22, H54.3, H54.8 |
| Traumatic brain injury | F07.0, F07.81, F07.89, F48.2, S04.011S, S04.012S, S04.019S, S04.02XS, S04.031S, S04.032S, S04.039S, S04.041S, S04.042S, S04.049S, S04.10XS, S04.11XS, S04.12XS, S04.20XS, S04.21XS, S04.22XS, S04.30XS, S04.31XS, S04.32XS, S04.40XS, S04.41XS, S04.42XS, S04.50XS, S04.51XS, S04.52XS, S04.60XS, S04.61XS, S04.62XS, S04.70XS, S04.71XS, S04.72XS, S04.811S, S04.812S, S04.819S, S04.891S, S04.892S, S04.899S, S04.9XXS, S06.0X0S, S06.0X1S, S06.0X2S, S06.0X3S, S06.0X4S, S06.0X5S, S06.0X6S, S06.0X7S, S06.0X8S, S06.0XAS, S06.0X9S, S06.1X0S, S06.1X1S, S06.1X2S, S06.1X3S, S06.1X4S, S06.1X5S, S06.1X6S, S06.1X7S, S06.1X8S, S06.1XAS, S06.1X9S, S06.2X0S, S06.2X1S, S06.2X2S, S06.2X3S, S06.2X4S, S06.2X5S, S06.2X6S, S06.2X7S, S06.2X8S, S06.2XAS, S06.2X9S, S06.300S, S06.301S, S06.302S, S06.303S, S06.304S, S06.305S, S06.306S, S06.307S, S06.308S, S06.30AS, S06.309S, S06.310S, S06.311S, S06.312S, S06.313S, S06.314S, S06.315S, S06.316S, S06.317S, S06.318S, S06.31AS, S06.319S, S06.320S, S06.321S, S06.322S, S06.323S, S06.324S, S06.325S, S06.326S, S06.327S, S06.328S, S06.32AS, S06.329S, S06.330S, S06.331S, S06.332S, S06.333S, S06.334S, S06.335S, S06.336S, S06.337S, S06.338S, S06.33AS, S06.339S, S06.340S, S06.341S, S06.342S, S06.343S, S06.344S, S06.345S, S06.346S, S06.347S, S06.348S, S06.34AS, S06.349S, S06.350S, S06.351S, S06.352S, S06.353S, S06.354S, S06.355S, S06.356S, S06.357S, S06.358S, S06.35AS, S06.359S, S06.360S, S06.361S, S06.362S, S06.363S, S06.364S, S06.365S, S06.366S, S06.367S, S06.368S, S06.36AS, S06.369S, S06.370S, S06.371S, S06.372S, S06.373S, S06.374S, S06.375S, S06.376S, S06.377S, S06.378S, S06.37AS, S06.379S, S06.380S, S06.381S, S06.382S, S06.383S, S06.384S, S06.385S, S06.386S, S06.387S, S06.388S, S06.38AS, S06.389S, S06.4X0S, S06.4X1S, S06.4X2S, S06.4X3S, S06.4X4S, S06.4X5S, S06.4X6S, S06.4X7S, S06.4X8S, S06.4XAS, S06.4X9S, S06.5X0S, S06.5X1S, S06.5X2S, S06.5X3S, S06.5X4S, S06.5X5S, S06.5X6S, S06.5X7S, S06.5X8S, S06.5XAS, S06.5X9S, S06.6X0S, S06.6X1S, S06.6X2S, S06.6X3S, S06.6X4S, S06.6X5S, S06.6X6S, S06.6X7S, S06.6X8S, S06.6XAS, S06.6X9S, S06.810S, S06.811S, S06.812S, S06.813S, S06.814S, S06.815S, S06.816S, S06.817S, S06.818S, S06.81AS, S06.819S, S06.820S, S06.821S, S06.822S, S06.823S, S06.824S, S06.825S, S06.826S, S06.827S, S06.828S, S06.82AS, S06.829S, S06.8A0S, S06.8A1S, S06.8A2S, S06.8A3S, S06.8A4S, S06.8A5S, S06.8A6S, S06.8AAS, S06.8A9S, S06.890S, S06.891S, S06.892S, S06.893S, S06.894S, S06.895S, S06.896S, S06.897S, S06.898S, S06.89AS, S06.899S, S06.9X0S, S06.9X1S, S06.9X2S, S06.9X3S, S06.9X4S, S06.9X5S, S06.9X6S, S06.9X7S, S06.9X8S, S06.9XAS, S06.9X9S, S06.A0XS, S06.A1XS |

*eTable 2. Lab measurements included as predictors*

| Lab measurements |
| --- |
| Creatinine |
| Calcium |
| Albumin |
| Ferritin |
| Hemoglobin A1C (HGBA1C) |
| Testosterone |
| Iron |
| Blood urea nitrogen |
| Homocysteine |
| Sodium |
| Alanine Aminotransferase |
| Total Protein |
| Total Bilirubin |
| Hematocrit |
| White Blood Cell Count |
| Platelet Count |
| High-Density Lipoprotein |
| Cholesterol |
| Triglycerides |
| Cholesterol/HDL Ratio |
| Thyroid Stimulating Hormone |
| Absolute Neutrophil Count |
| Absolute Lymphocyte Count |
| Prostate-Specific Antigen |
| Globulin |
| Total Iron-Binding Capacity |
| Iron Saturation |
| Microalbumin |
| C-Reactive Protein |
| Free Triiodothyronine |
| Amylase |
| Total Thyroxine |
| Prolactin |

*eTable 3*

| Class | RxClass: Classification of drugs by ATC1-4 nomenclature |
| --- | --- |
| Blood Pressure Medications | |
| Antihypertensives | 2679059, 2631887, 1442132, 1439816, 358274, 358263, 266604, 214620, 214418, 142432, 91240, 82027, 75207, 49276, 40114, 9895, 9260, 8629, 7476, 6984, 6876, 6673, 5487, 5470, 2599, 2409 |
| Diuretics | 644, 214212, 142424, 1808, 2396, 2409, 302285, 1294548, 298869, 62349, 4108, 4109, 2562811, 4603, 5487, 324042, 258337, 5764, 6860, 6916, 9997, 358257, 38413, 10763 |
| Antiadrenergic agents, centrally acting | 2599, 142432, 40114, 6876, 9260 |
| Agents acting on the renin-angiotensin system | 325646, 802519, 17767, 214223, 1008801, 852897, 1009015, 1033889, 901212, 729455, 1091643, 1235143, 1091642, 18867, 214287, 235758, 214354, 284628, 135481, 1998, 214357, 2409, 3827, 214536, 203123, 3829, 83515, 352375, 50166, 261415, 227278, 5487, 214617, 214618, 214619, 214622, 817496, 261417, 284636, 214626, 83818, 29046, 52175, 203160, 30131, 236066, 31555, 1798280, 321064, 118463, 54552, 1600711, 72260, 35208, 35296, 1656328, 1656339, 73494, 38454, 214866, 69749, 11170, 203138 |
| Beta blocking agents | 149, 1202, 151195, 1369, 214288, 1520, 142144, 19484, 214317, 142146, 20352, 668310, 2409, 49737, 203222, 5487, 214621, 214623, 6185, 202693, 6918, 221124, 203191, 7226, 31555, 8332, 8787, 82084, 9947, 7008, 10600 |
| Calcium channel blockers | 17767, 2047714, 140587, 233603, 3443, 203211, 4316, 33910, 2376944, 7396, 235230, 7417, 7426, 7435, 11170, 203138 |
| Cholesterol meds | |
| Statins | 83367, 41127, 6472, 861634, 42463, 301542, 36567 |
| Bile acid sequestrants | 2447, 141626, 2685 |
| Cholesterol absorption inhibitors | 341248 |
| Nicotinic acid (niacin) | 7393 |
| PCSK9 inhibitors | 1659152, 1665684, 2588243 |
| Fibrates | 8703, 4719, 1525 |
| Psychotropic meds | |
| SSRI antidepressants | 2556, 321988, 4493, 42355, 32937, 36437 |
| SNRI antidepressants | 734064, 72625, 1433212, 588250, 39786 |
| Tricyclic antidepressants | 704, 722, 2597, 3247, 3634, 3638, 5691, 6465, 6646, 7531, 7674, 8886, 10834 |
| Anticonvulsants | 21241, 2598, 2353, 3322, 6470, 1739745, 2045371, 2002, 2265690, 4135, 24812, 25480, 623400, 28439, 114477, 32624, 1356552, 8134, 8183, 72236, 187832, 8691, 69036, 2054968, 31914, 38404, 40254, 14851, 39998 |
| Sedative hypnotics | 2591497, 461016, 2272403, 596205, 1547099, 74667, 39993 |
| Inhaled anesthetics | 7486, 5095, 6026, 36453, 27340 |
| Barbiturates | 719, 6847, 8004, 8134, 8691, 10493 |
| Benzodiazepines | 596, 1749, 19790, 2356, 21241, 2598, 2353, 2622, 3322, 4077, 4501, 28181, 6470, 28894, 6680, 6960, 7440, 7781, 8627, 35185, 10355, 37985, 38365, 10767 |
| Anxiolytics | 1827, 8332, 35623 |
| Atypical antipsychotics | 46303, 89013, 1673265, 784649, 1658314, 1667655, 2626, 73178, 2275602, 1040028, 29961, 61381, 679314, 1791685, 51272, 35636, 115698 |
| Conventional antipsychotics | 2403, 3648, 4495, 4496, 5093, 6852, 6475, 7019, 8766, 8076, 8331, 10502, 10510, 10800, 114176 |
| Cholinesterase inhibitors | 2690627, 135447, 4637, 183379 |
| Memantine | 6719 |
| Lithium | 6448 |
| Bupropion | 42347 |
| Anticoagulants | |
| Anticoagulants | 11289, 5224, 67109, 78484,67108, 69646, 321208 |

*eTable 4. Predictors’ estimates from the univariate analysis.*

| Predictor | RR (95% CI) | P-value |
| --- | --- | --- |
| Chronic Pain | 1.41 (1.26-1.57) | <0.001 |
| BP medications | 1.17 (1.11-1.23) | <0.001 |
| Albumin | 1.20 (1.18-1.23) | <0.001 |
| Hospital visits | 1.03 (1.01-1.06) | <0.001 |
| BUN | 1.05 (1.02-1.07) | <0.001 |
| HbA1c | 0.94 (0.91-0.96) | <0.001 |
| Sodium | 0.91 (0.89-0.93) | <0.001 |
| Missing TSH | 0.86 (0.81-0.90) | <0.001 |
| Anticoagulants | 0.71 (0.67-0.74) | <0.001 |
| Anxiety | 0.64 (0.61-0.67) | <0.001 |
| Missing HDL | 0.63 (0.57-0.69) | <0.001 |
| Black race | 0.83 (0.78-0.88) | <0.001 |
| Age | 0.78 (0.77-0.80) | <0.001 |
| Medicaid | 0.63 (0.59-0.68) | <0.001 |
| Stroke | 0.63 (0.60-0.66) | <0.001 |
| Alcohol use disorder | 0.58 (0.51-0.65) | <0.001 |
| Depression | 0.51 (0.48-0.53) | <0.001 |
| Bipolar Disorder | 0.42 (0.38-0.46) | <0.001 |
| ADHD | 0.39 (0.33-0.47) | <0.001 |
| Psychotropic medications | 0.29 (0.26-0.31) | <0.001 |
| Schizophrenia | 0.33 (0.29-0.36) | <0.001 |
| Parkinson’s Disease | 0.34 (0.32-0.37) | <0.001 |


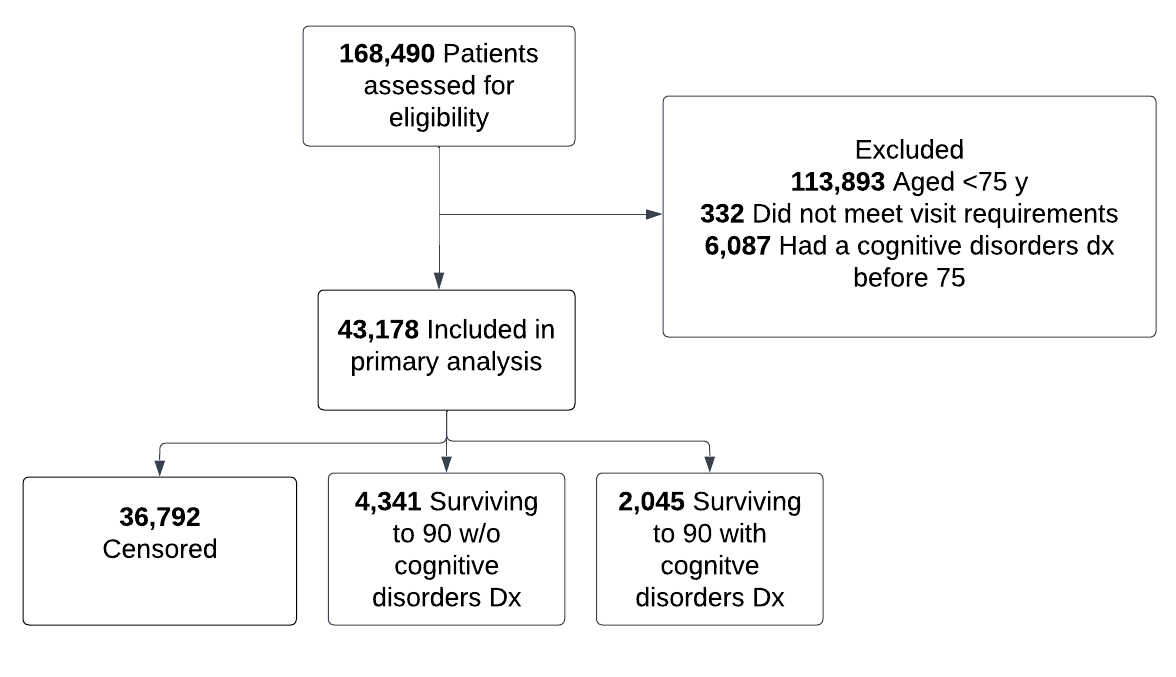


*eFigure 1. Waterfall Diagram showing the inclusion criteria and the two cohorts of people surviving to 90.*

*
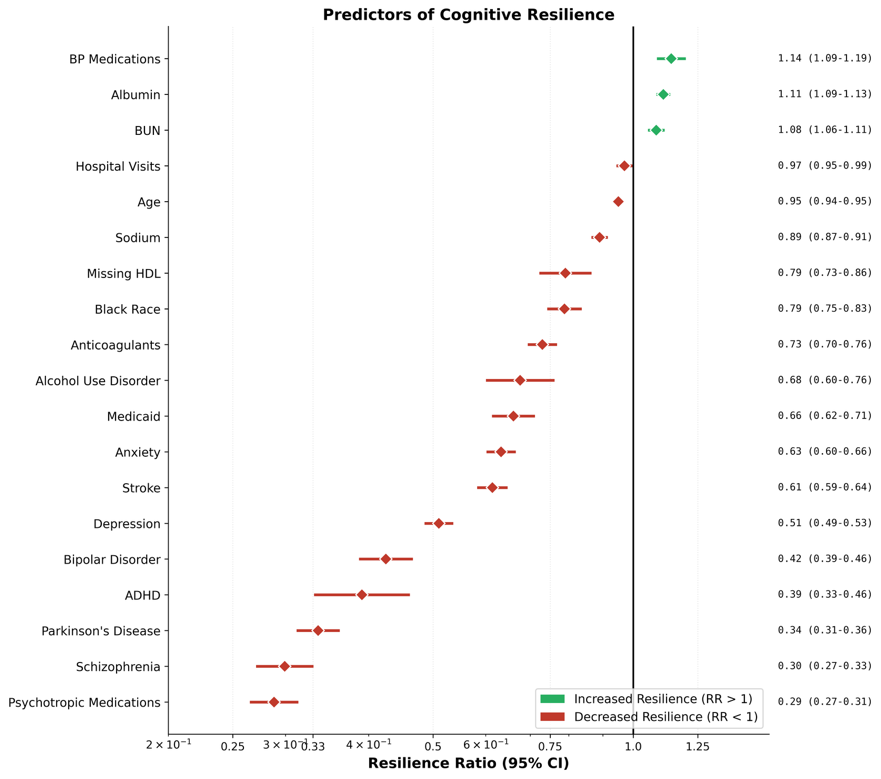
*

*eFigure 2. All statistically significant predictors from Fine-Gray analysis.*

*
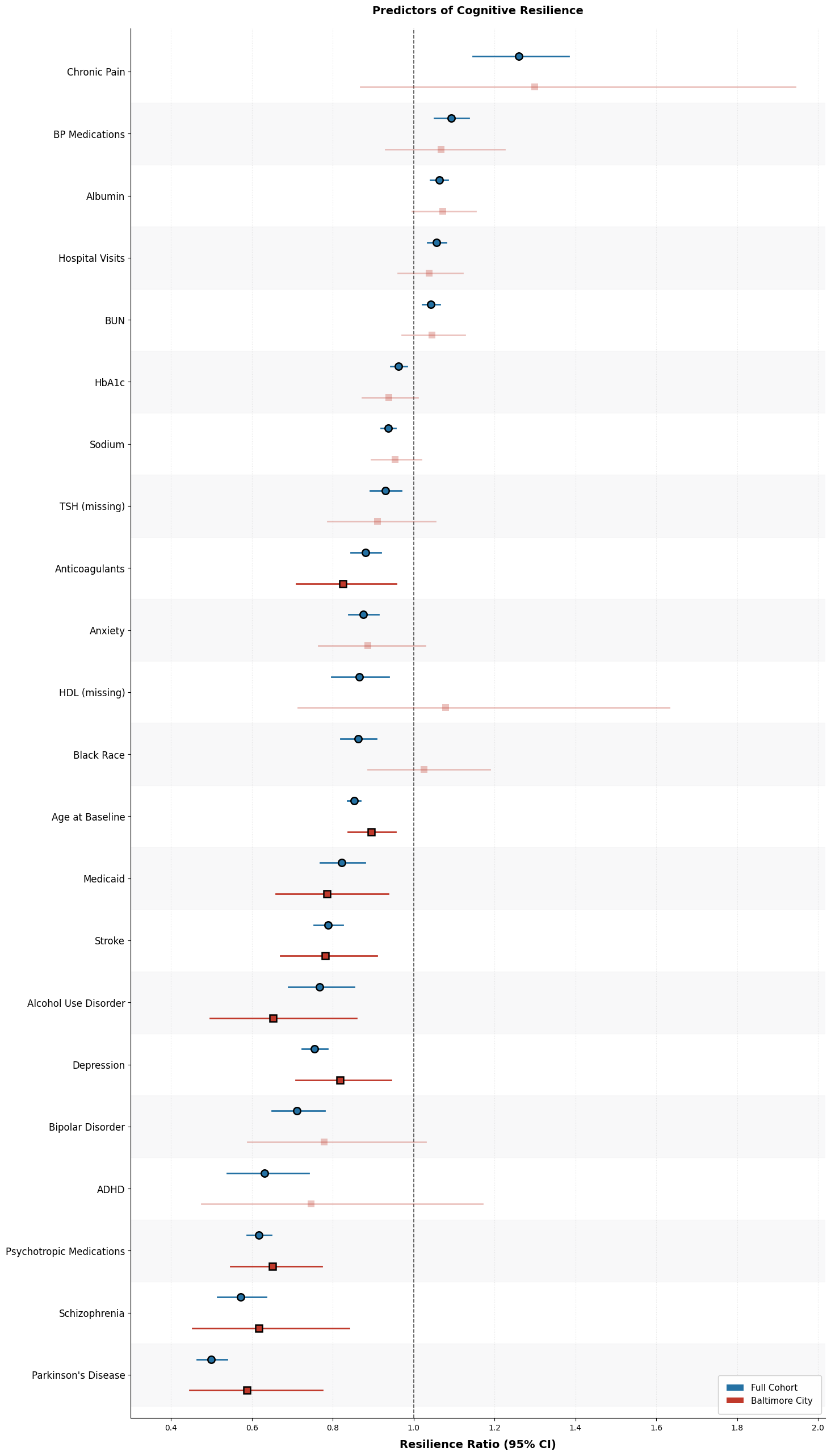
*

*eFigure 3. Predictors of cognitive resilience in the full cohort (in blue) and Baltimore residents subsample (in red) as part of the sensitivity analysis. Predictors with RR>1 are associated with increased resilience and predictors with RR<1 with decreased resilience. Predictors with faded lines are not statistically significant.*
